# Overexpression of Grainyhead-like 3 causes spina bifida and interacts genetically with mutant alleles of Grhl2 and Vangl2 in mice

**DOI:** 10.1093/hmg/ddy313

**Published:** 2018-09-04

**Authors:** Sandra C P De Castro, Peter Gustavsson, Abigail R Marshall, William M Gordon, Gabriel Galea, Evanthia Nikolopoulou, Dawn Savery, Ana Rolo, Philip Stanier, Bogi Andersen, Andrew J Copp, Nicholas D E Greene

**Affiliations:** 1 Developmental Biology & Cancer Programme, UCL Great Ormond Street Institute of Child Health, University College London, London, UK; 2 Genetics and Genomic Medicine Programme, UCL Great Ormond Street Institute of Child Health, University College London, London, UK; 3 Department of Molecular Medicine and Surgery, Karolinska Institute, Stockholm, Sweden; 4 Department of Biological Chemistry, University of California Irvine, Irvine, California, USA; 5 Department of Medicine, School of Medicine, University of California Irvine, Irvine, California, USA

## Abstract

The genetic basis of human neural tube defects (NTDs), such as anencephaly and spina bifida (SB), is complex and heterogeneous. Grainyhead-like genes represent candidates for involvement in NTDs based on the presence of SB and exencephaly in mice carrying loss-of-function alleles of *Grhl2* or *Grhl3*. We found that reinstatement of *Grhl3* expression, by bacterial artificial chromosome (BAC)-mediated transgenesis, prevents SB in *Grhl3*-null embryos, as in the *Grhl3* hypomorphic *curly tail* strain. Notably, however, further increase in expression of *Grhl3* causes highly penetrant SB. *Grhl3* overexpression recapitulates the spinal NTD phenotype of loss-of-function embryos, although the underlying mechanism differs. However, it does not phenocopy other defects of *Grhl3*-null embryos such as abnormal axial curvature, cranial NTDs (exencephaly) or skin barrier defects, the latter being rescued by the Grhl3-transgene. Grhl2 and Grhl3 can form homodimers and heterodimers, suggesting a possible model in which defects arising from overexpression of Grhl3 result from sequestration of Grhl2 in heterodimers, mimicking *Grhl2* loss of function. This hypothesis predicts that increased abundance of Grhl2 would have an ameliorating effect in *Grhl3* overexpressing embryo. Instead, we observed a striking additive genetic interaction between *Grhl2* and *Grhl3* gain-of-function alleles. Severe SB arose in embryos in which both genes were expressed at moderately elevated levels that individually do not cause NTDs. Furthermore, moderate *Grhl3* overexpression also interacted with the *Vangl2^Lp^* allele to cause SB, demonstrating genetic interaction with the planar cell polarity signalling pathway that is implicated in mouse and human NTDs.

## Introduction

Although neural tube defects (NTDs) are among the most common birth defects worldwide and have a strong genetic component, the specific contributors to genetic risk are not well understood (1,4). Identifying the molecular determinants of human NTDs is hindered by their complex, multifactorial nature and likely heterogeneity between cases. Moreover, most cases are sporadic rather than familial and *de novo* mutations may also play a significant role in spina bifida (SB) causation (5,6). Patterns of recurrence risk support oligogenic or polygenic models in which most NTDs result from a combination of one or more genetic factors with contribution from environmental risk factors, both positive and negative (7). It is anticipated that large-scale whole-exome and whole-genome sequencing efforts will provide a greater understanding of the genetic basis of NTDs but analysis of these large data sets and assignment of causation to coding variants and/or potential regulatory mutations will not be trivial.

Candidate genes for human NTDs may be indicated by knowledge of environmental risk factors, such as folate status and maternal diabetes, and causative genes in genetic models of which several hundred have been identified in mice (2,8). Mouse models have demonstrated a crucial role for members of the *grainyhead-like* family of transcription factors in neural tube closure. *Grhl3*-null embryos develop fully penetrant SB (9,10) and a hypomorphic allele of *Grhl3* is the main genetic cause of SB in the *curly tail* strain (11). Each of these strains also exhibit a low frequency of exencephaly, which results from incomplete closure of the cranial neural tube and leads to anencephaly in late gestation.

Analysis of *Grhl3*-null and tissue-specific knockout embryos indicates that the initial defect leading to failure of spinal neurulation is localized to the surface ectoderm component of the closing neural folds, corresponding with its prominent early expression in this cell layer (12). *Grhl3* is also expressed in the node-streak border/caudo-lateral epiblast and, later and transiently, in the neuroepithelium as well as in the gut endoderm (9,11,12). Among these tissues, knockout of *Grhl3* in the gut endoderm causes spinal NTDs, but with later onset than in null embryos. These defects result from excess ventral curvature of the body axis, as in *curly tail* (*Grhl3^ct^*) mutant embryos (12,13). Hence, *Grhl3* deficit leads to tissue-specific abnormalities that inhibit closure at two successive stages of spinal neurulation.

The extent to which *GRHL3* mutations may contribute to human NTDs is not yet clear. Both *de novo* and rare inherited missense mutations of *GRHL3* have been reported in SB cases at a frequency that suggests a role in determining NTD predisposition (6,14). *GRHL3* mutations have also been reported in individuals with cleft lip and palate and individuals with syndromic (Van der Woude syndrome) and non-syndromic isolated cleft palate (15,17), consistent with *GRHL3* expression in the oral ectoderm*.* Sharing of a missense mutation in independent SB and cleft palate cases supports the concept that *GRHL3* may contribute to both defects (6,15).

The potential for regulatory mutations in *GRHL3* to contribute to causation of human NTDs has been largely unexplored to date. In mice, a regulatory mutation likely underlies the diminished expression of *Grhl3*, which causes SB in the *curly tail* mouse (11). Tissues from human fetuses with NTDs have revealed hypomethylation of CpG islands within the 5′UTR and introns of *GRHL3* (18), perhaps suggesting that *GRHL3* misexpression may lead to NTDs. In the current study we examined this possibility in mouse models. Notably, we found that overexpression of *Grhl3* causes a high frequency of severe SB. Moreover, even moderately elevated abundance of *Grhl3* was found to cause SB when in combination with mutant alleles of other NTD genes: *Grhl2* or *Vangl2*. Hence, insufficient or excess levels of *Grhl3* can both cause SB.

## Results

### Overexpression of *Grhl3* causes NTDs

**Figure 1:**
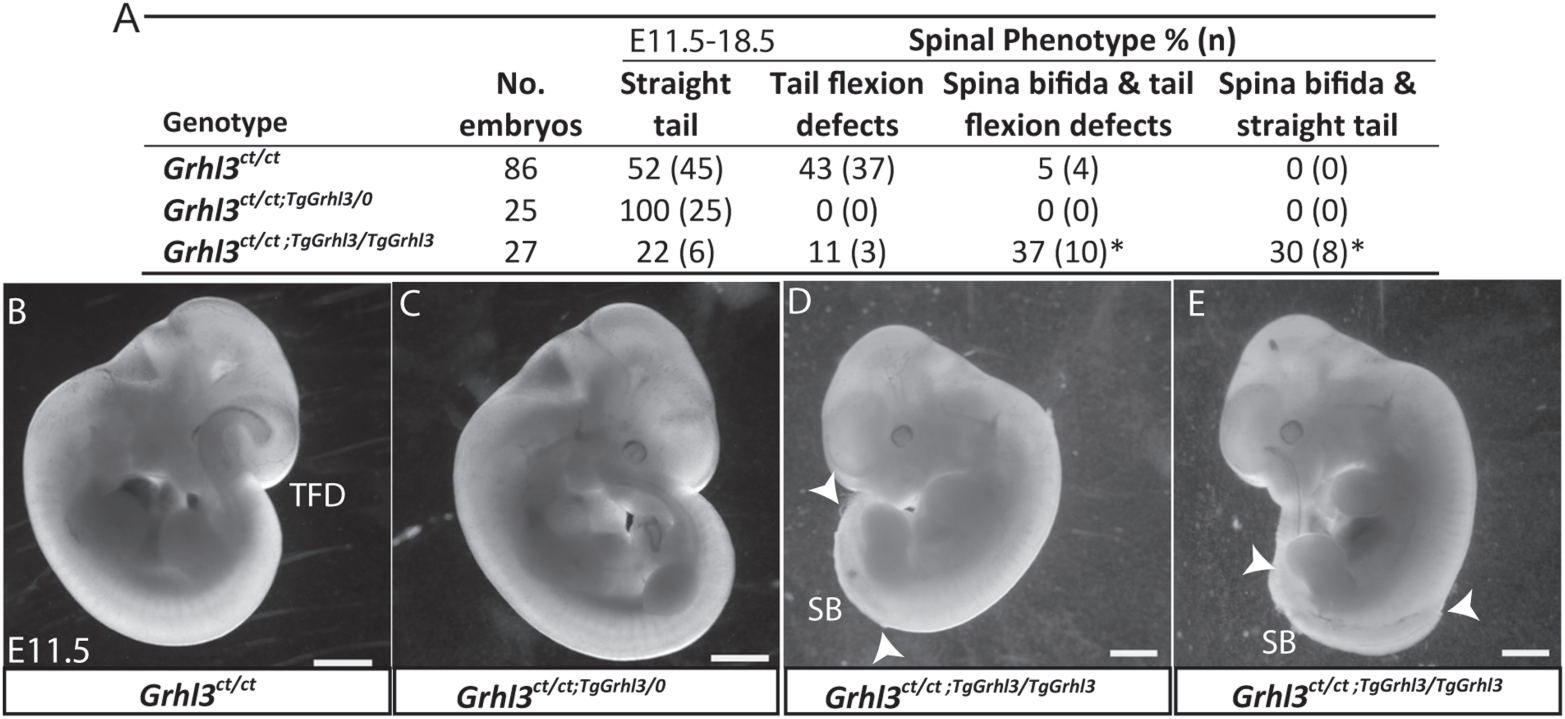
Spinal NTDs in *Grhl3*-transgenic mice. (**A**) Occurrence of SB among offspring of matings between *Grhl3*^*ct/ct*;*TgGrhl3/0*^ (BAC-hemizygous) and *Grhl3^ct/ct^* mice analysed at E11.5–18.5 (*, significant difference from other genotypes; *P* < 0.001, Chi-square). (**B–E**) Embryos of the three genotypes at E11.5. All *Grhl3*^*ct/ct*;*TgGrhl3/0*^ embryos appeared normal with a straight caudal region (C), whereas a proportion of *Grhl3^ct/ct^* (B) and *Grhl3*^*ct/ct*;*TgGrhl3/TgGrhl3*^ (D, E) exhibited TFDs and/or SB (extent of open region indicated by arrowheads). Scale bar represents 1 mm.

Reinstatement of *Grhl3* expression, mediated by bacterial artificial chromosome (BAC) transgenesis, prevents spinal NTDs in *curly tail* mice that have partial loss of function of *Grhl3* (11). We investigated the potential effect of increasing levels of *Grhl3* expression by intercross of hemizygous *Grhl3* BAC-transgenic mice (*Grhl3*^*ct/ct*;*TgGrhl3/0*^) to generate litters that include embryos carrying the BAC in homozygosity (*Grhl3*^*ct/ct*;*TgGrhl3/TgGrhl3*^). Litters were genotyped by BAC-specific polymerase chain reaction (PCR) and quantitative genomic PCR (GqPCR). As in our previous study (11), *Grhl3*^*ct/ct*;*TgGrhl3/0*^ embryos (i.e. *ct/ct* embryos also hemizygous for the *Grhl3-*BAC) did not display spinal NTDs, whereas a proportion of *Grhl3^ct/ct^* embryos developed SB and/or tail flexion defects (TFDs) (Fig.1). Remarkably, we observed SB in 67% of *Grhl3*^*ct/ct*;*TgGrhl3/TgGrhl3*^ embryos, suggesting that overexpression of *Grhl3* prevents neural tube closure (Fig. 1).

**Figure 2:**
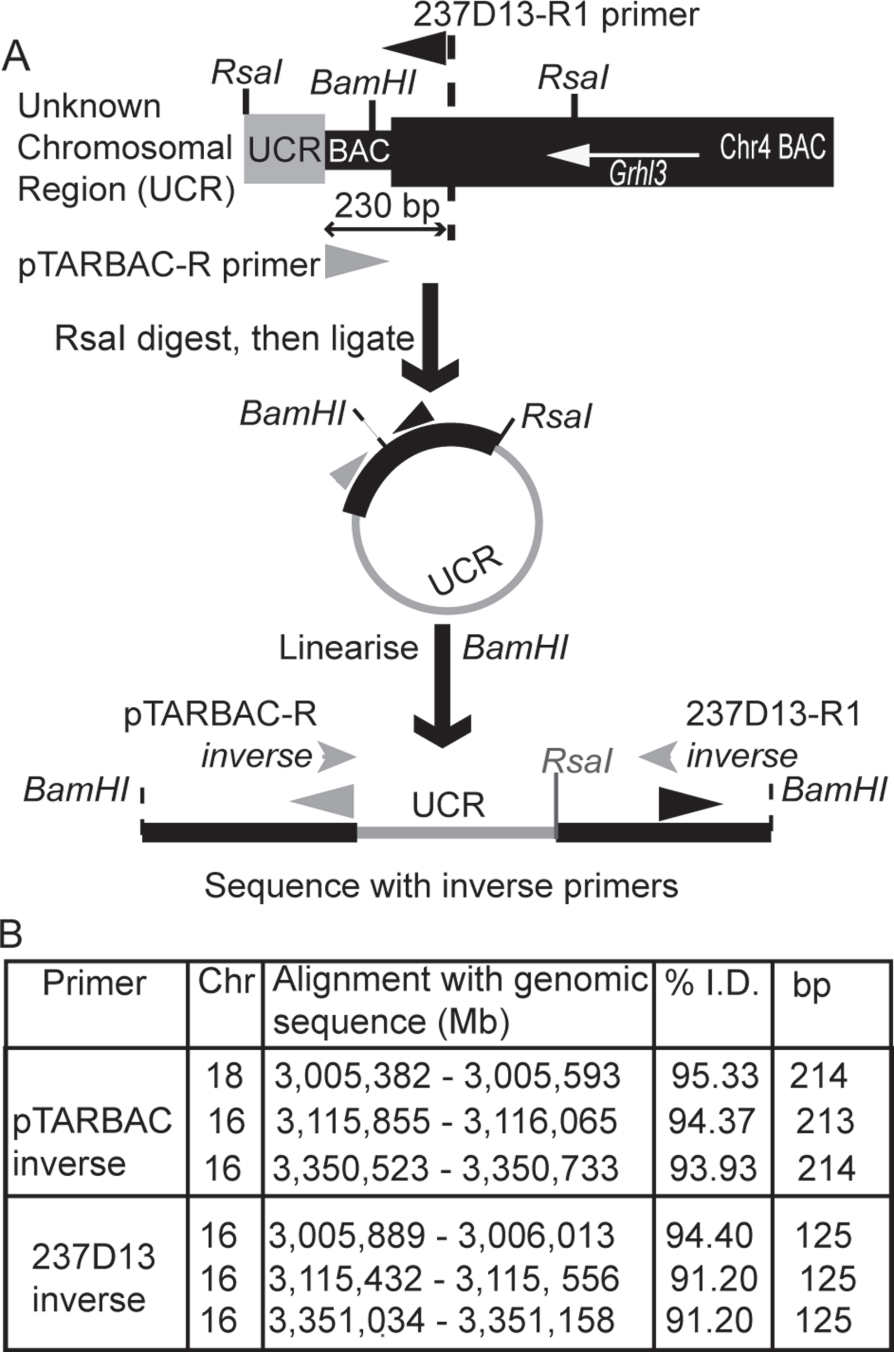
Localization of BAC-transgene in *Grhl3*^*ct/ct*;*TgGrhl3/0*^ embryos. (**A**) Inverse PCR was used to isolate genomic fragments adjacent to the insertion site of the *Grhl3-*containing BAC. (**B**) Sequence tags most closely aligned to the reference genomic sequence in a region on chromosome 18 with insertion of the BAC at 18: 3,005,382 in a low complexity repeat. Inverse PCR fragments also show homology to regions on chromosome 16 but with lower identity.

The NTD phenotype of *Grhl3* overexpressing embryos could theoretically result from homozygous insertion of the transgene into an essential endogenous locus. To investigate this, the genomic location of the BAC was determined using inverse PCR (Fig. 2A and B). Sequence analysis indicated that the transgene insertion site is at position 3,005,382 on chromosome 18, in a low-complexity region. This was confirmed by PCR amplification of genomic DNA, using a series of primer pairs located in the transgene and putative chromosome 18 location (Fig. 2C and Supplementary Material, Fig. S1A). This site is more than 100 kb from the nearest recognized gene (*vmn1r238*) and 266 kb from the nearest gene (*Crem*) that has detectable embryonic expression at E10.5 (Supplementary Material, Fig. S1B). Fluorescence *in situ* hybridization (FISH) analysis on whole blood cultures confirmed that the BAC is present in only one location (Supplementary Material, Fig. S1D and E). We conclude that insertional mutagenesis is very unlikely to explain the NTDs observed in *Grhl3* overexpressing embryos.

Further evidence that *Grhl3* overexpression causes NTDs was provided by backcross of the *Grhl3* transgene onto a wild-type BALB/c genetic background. NTDs did not occur among wild-type embryos (n = 20), but among hemizygous (+/+*^TgGrhl3/0^*) embryos we observed a low frequency of SB or TFDs (3/20; 15%). These defects are predicted to arise because the level of endogenous *Grhl3* expression from the wild-type allele is higher than in the hypomorphic *ct* strain such that overexpression mediated by a single copy of the BAC is sufficient to exceed the level that is compatible with neural tube closure. Consistent with this, all homozygous (+/+*^TgGrhl3/TgGrhl3^*) embryos developed SB on this genetic background (18/18; 100%).

### SB in *Grhl3* overexpressing embryos results from early failure of posterior neuropore closure

**Figure 3:**
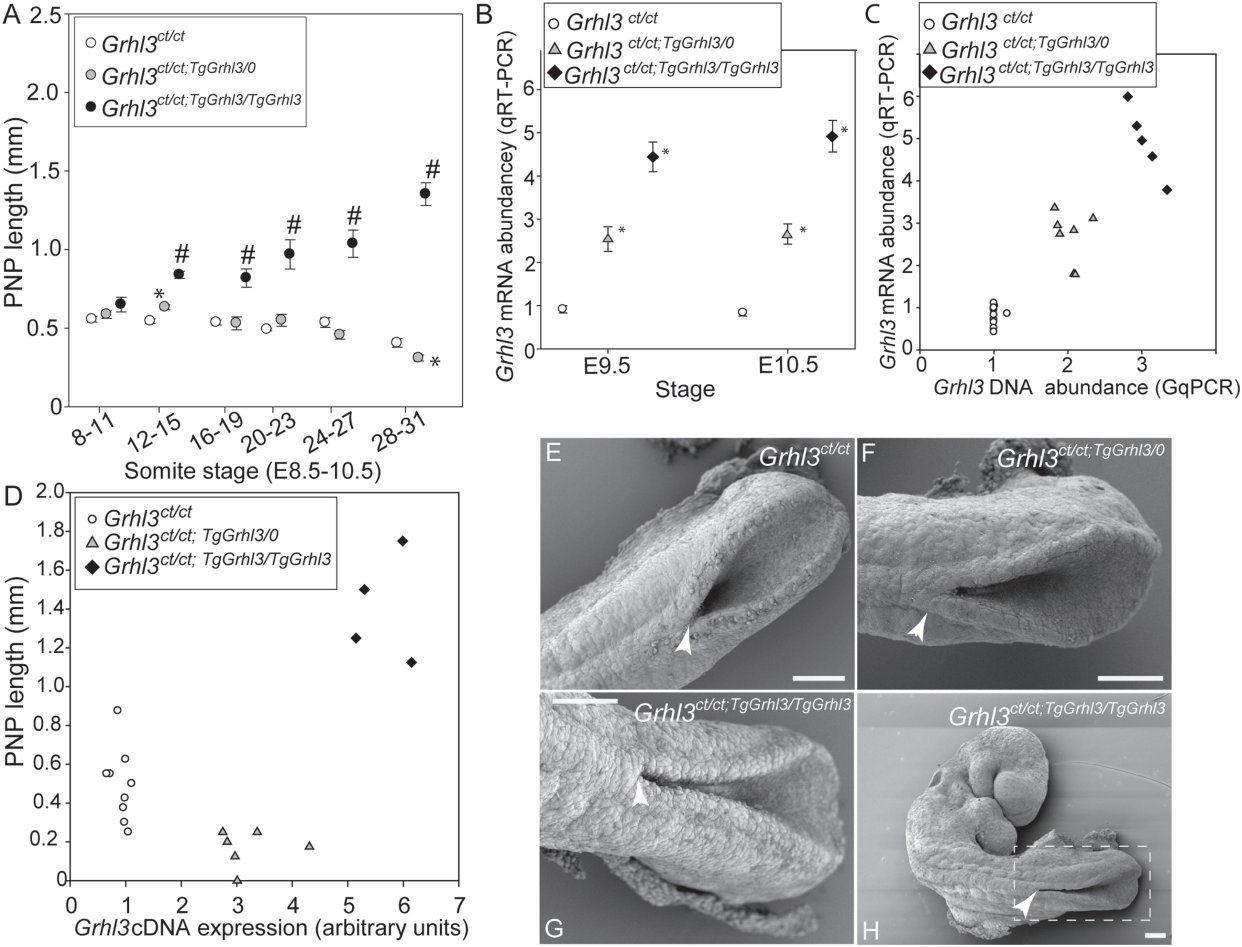
Excess expression of *Grhl3* results in spinal NTDs owing to failure of PNP closure. (**A**) Among litters from *Grhl3*^*ct/ct*;*TgGrhl3/0*^ matings analysed at E8.5–10.5, the PNP was significantly enlarged among *Grhl3*^*ct/ct*;*TgGrhl3/TgGrhl3*^ embryos (n = 64) from the 12–15 somite stage onwards (#, significant difference from both other genotypes, *P* < 0.001). PNP closure was normalized in *Grhl3*^*ct/ct*;*TgGrhl3/0*^ embryos (n = 123) compared with *Grhl3^ct/ct^* littermates (n = 176) at 28–31 somites (^*^, significantly different from *Grhl3^ct/ct^*, *P* < 0.001). Mean ± SEM values; n = 4–77 embryos/genotype/stage (see Supplementary, Fig. S2 for plot of individual data). (**B**) Abundance of *Grhl3* mRNA varies significantly with genotype at E9.5 and E10.5 (^*^P < 0.0001 ANOVA; Holm-Sidak pairwise analysis). For context, we previously found that *Grhl3* expression in *ct/ct* embryos at E10.5 was ∼50% of that in partially congenic wild-type (+*^ct^*) embryos (11). (**C**) Analysis of individual embryos at E10.5 (28–29 somites) shows that increased abundance of *Grhl3* genomic DNA (in transgenic embryos) results in gene dosage-dependent increase in *Grhl3* mRNA expression. qG-PCR signal corresponds to 2, 3 and 4 copies of *Grhl3* in *ct/ct*, hemizygous and homozygous transgenics, respectively. (**D**) Moderate overexpression of *Grhl3* normalizes PNP closure in individual *Grhl3*^*ct/ct*;*TgGrhl3/0*^ embryos, compared with *Grhl3^ct/ct^*, whereas excess expression prevents PNP closure, in *Grhl3*^*ct/ct*;*TgGrhl3/TgGrhl3*^. (**E–H**) Scanning electron micrographs at E9 (13 somite stage) show typical appearance of the closing PNP. Neural folds are elevated and apposed in all three genotypes (E–G) but failure of closure progression is already apparent in *Grhl3*^*ct/ct*;*TgGrhl3/TgGrhl3*^ embryos (G,H), leaving a narrow unclosed region (compare region adjacent to white arrow E–H). Scale bars represent 0.1 mm.

In addition to a higher frequency of SB than in *Grhl3^ct/ct^* embryos, the size of the open lesion was also greater in *Grhl3* overexpressing embryos at E11.5–13.5 (Fig. 1 and Supplementary Material, Fig. S2A–D). Previous studies showed that the posterior neuropore (PNP) length of *Grhl3^ct/ct^* embryos becomes larger than genetically matched wild-type embryos from the 25–27somite stage (E10.5) (11). In order to assess the stage at which spinal neurulation fails in *Grhl3* overexpressing embryos, we collected litters generated by intercross of *Grhl3*^*ct/ct*;*TgGrhl3/0*^ mice. Among embryos analysed at E9–10.5, the PNP length was already larger among *Grhl3*^*ct/ct*; *TgGrhl3/TgGrhl3*^ embryos than *Grhl3^ct/ct^* littermates from the 12–15 somite stage (E9) onwards (Fig. 3A and Supplementary Material, Fig. S2E). This earlier failure of closure explains why the extent of the open SB lesion is greater among *Grhl3* overexpressing fetuses than in their hypomorphic littermates.

Among embryos at E9.5 and E10.0, *Grhl3* mRNA was upregulated by 2–3-folds in the caudal region of *Grhl3*^*ct/ct*; *TgGrhl3/0*^ embryos and 4–6-folds in *Grhl3*^*ct/ct*; *TgGrhl3/TgGrhl3*^, compared with *Grhl3^ct/ct^* (Fig. 3B). This equates to ∼1.3–1.5- and 2.5–3-fold higher expression than in wild-type embryos with a similar genetic background (based on our finding that *Grhl3* abundance in *ct/ct* embryos is ∼50% of that in partially congenic (+*^ct^*/+*^ct^*) wild types (11). Hence, *Grhl3* mRNA abundance (by qRT-PCR) correlated with the number of copies of the *Grhl3* gene, determined by GqPCR (Fig. 3C). We conclude that during spinal neurulation the PNP becomes enlarged in *Grhl3^ct/ct^* hypomorphic embryos compared with *Grhl3*^*ct/ct*; *TgGrhl3/0*^ embryos that have mildly elevated *Grhl3* expression, but a further increase in *Grhl3* expression results in an even larger PNP in *Grhl3*^*ct/ct*; *TgGrhl3/TgGrhl3*^ embryos (Fig. 3D), resulting in SB. Scanning electron microscopy showed that the enlarged PNP of *Grhl3* overexpressing embryos was characteristically very narrow (compare Fig. 3E–F with Fig. 3G–H), suggesting that NTDs do not result from a defect in elevation or bending of the neural folds.

### Balance between excess and insufficient expression of *Grhl3* in spinal neurulation

Homozygous embryos for *Grhl3*-null (*Grhl3^-/-^*) or gain-of-function (*Grhl3*^*ct/ct*; *TgGrhl3/TgGrhl3*^) alleles develop severe spinal NTDs. In order to further investigate the correlation between *Grhl3* expression level and neural tube closure we asked whether transgenic expression was sufficient to rescue *Grhl3*-null NTDs and/or vice versa. *Grhl3^+/-^* and *Grhl3*^*ct/ct*;*TgGrhl3/0*^ mice were crossed and offspring with genotype *Grhl3*^*ct/-*; *TgGrhl3/0*^ were intercrossed to generate experimental litters carrying combinations of the null allele and the *Grhl3-*BAC (Table 1). *Grhl3^-/-^* and *Grhl3*^*ct/ct*; *TgGrhl3/TgGrhl3*^ fetuses developed SB as expected. The majority of embryos with *Grhl3^ct/-^* genotype exhibited TFDs, with SB also present in 50% (5 of 10) of embryos, intermediate between that normally observed in *Grhl3^ct/ct^* hypomorphs and *Grhl3*^-/-^ embryos. Hemizygosity for the transgene normalized neural tube closure irrespective of the endogenous *Grhl3* genotype, with no NTDs arising in *Grhl3^ct/ct;TgGrhl3/0^* or *Grhl3^ct/-;TgGrhl3/0^* embryos, or most notably in *Grhl3^-/-;TgGrhl3/0^* that lacks endogenous *Grhl3* (Table 1). SB occurred in only three of eight homozygous null embryos that were also homozygous for the *Grhl3-*BAC (*Grhl3^-/-;TgGrhl3/TgGrhl3^*; Table 1). Overall, these findings suggest that overexpression of *Grhl3* is sufficient to rescue the null phenotype and that nullizygosity for *Grhl3* can partially rescue NTDs resulting from *Grhl3* overexpression, highlighting the importance of *Grhl3* gene dosage.

**Table 1:** Phenotypes of embryos carrying combinations of *Grhl3* loss of function and overexpressing alleles

		**Spinal phenotype % (n)**			
Genotype	**No. embryos**	**Straight tail**	**TFDs**	**SB** **& TFDs**	***P***
*Grhl3^ct/ct^*	13	77 (10)	15 (2)	8 (1)	
*Grhl3* ^*ct/ct*;*TgGrhl3/0*^	8	100 (8)	0 (0)	0 (0)	
*Grhl3* ^*ct/ct*;*TgGrhl3/TgGrhl3*^	9	11 (1)	11 (1)	78 (7)	<0.001
*Grhl3^ct/-^*	10	20 (2)	30 (3)	50 (5)	
*Grhl3* ^*ct/-*;*TgGrhl3/0*^	10	100 (12)	0 (0)	0 (0)	
*Grhl3* ^*ct/-*;*TgGrhl3/TgGrhl3*^	10	30 (3)	0 (0)	70 (7)	<0.001
*Grhl3^-/-^*	11	0 (0)	0 (0)	100 (11)	
*Grhl3* ^*-/-*;*TgGrhl3/0*^	12	100 (12)	0 (0)	0 (0)	
*Grhl3* ^*-/-*;*TgGrhl3/TgGrhl3*^	8	62 (5)	0 (0)	38 (3)	<0.001

Offspring of *Grhl3*^*ct/-*;*TgGrhl3/0*^ intercrosses were assessed at E11.0–18.5 for the presence of NTDs and/or TFDs. NTDs did not arise among embryos heterozygous for the *Grhl3* transgene irrespective of the endogenous genotype. Differences in the number (0, 1 or 2) of *Grhl3* transgene copies was associated with significant variation in the distribution of spinal phenotypes among *Grhl3^ct/ct^*, *Grhl3^ct/-^* and *Grhl3^-/-^* embryos (*P* < 0.001; Chi-square).

**Figure 4:**
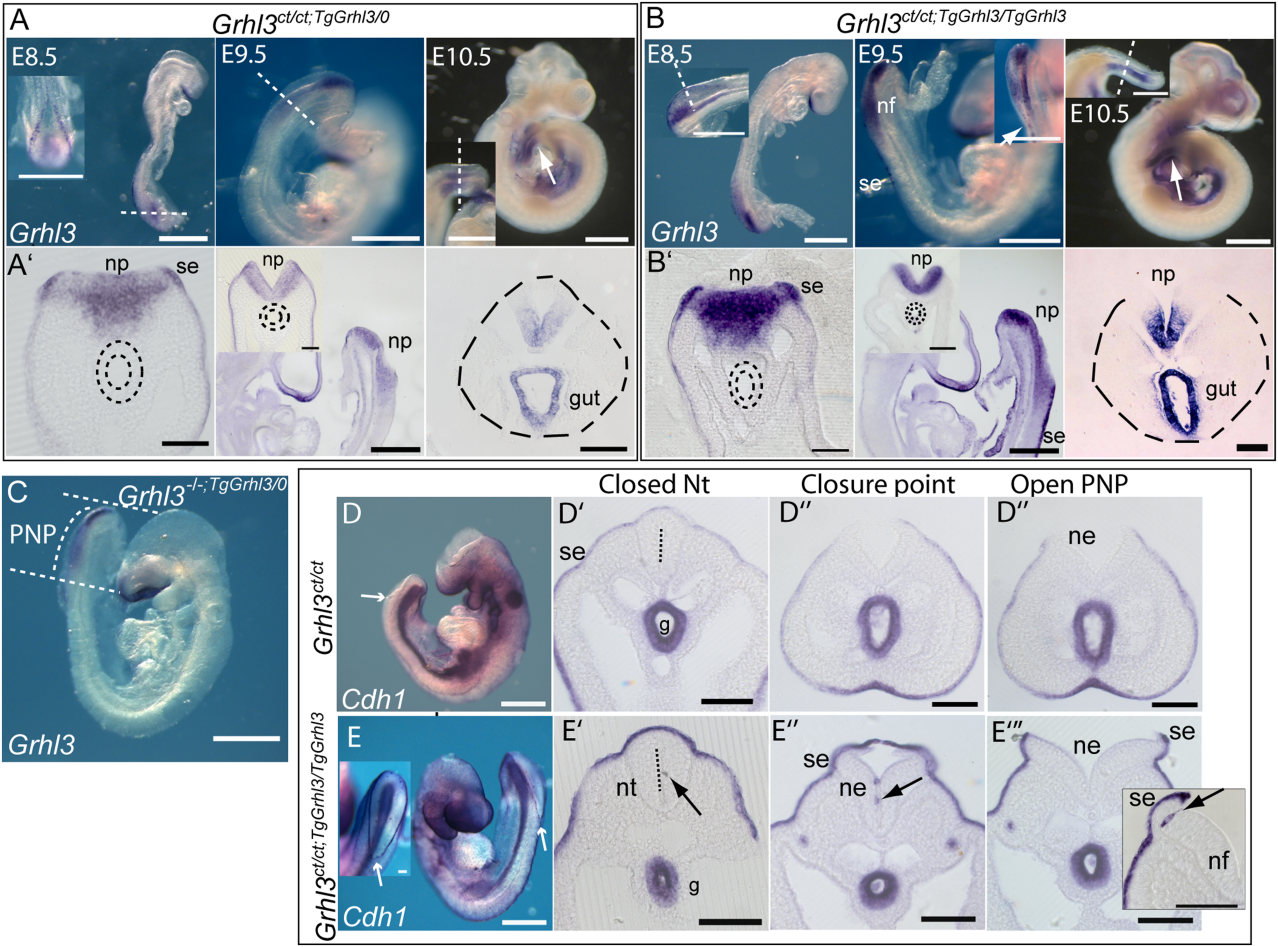
Overexpression of *Grhl3* and *Cdh1* in Grhl3-transgenic embryos. (**A and B**) *Grhl3* expression in hemizygous (A) and homozygous (B) *Grhl3*-BAC transgenics detected by WMISH. Expression is detected in the expected domains in the surface ectoderm (se), neural plate (np) and hindgut (arrows in whole mounts; dotted lines in sections). Transverse sections are at the level of white dotted line on corresponding whole mount images; sagittal sections are shown at E9.5. (**C**) On a *Grhl3*-null background, the expression pattern of *Grhl3* (entirely from the transgene), resembles the previously reported endogenous expression pattern. (**D and E**) *Cdh1* shows the expected expression in surface ectoderm (se) and gut endoderm (g) in *Grhl3^ct/ct^* embryos (D). In *Grhl3*-transgenic embryos (E), *Cdh1* expression appears more intense in the surface ectoderm (se) and occasional *Cdh1*-postive cells (arrows in E’-E”’) are present in the recently closed neural tube (nt; L) and the neuroepithelial component (ne) of the open neural folds (E”’, inset). Figure shows representative embryos at E9.5 (19–20 somite stage; D,E) with site of neural fold closure shown by white arrow in whole mounts. Scale bar = 0.5 mm in whole mounts; 0.1 mm in sections.

### Abnormal function of the surface ectoderm is implicated in causation of spinal NTDs in *Grhl3* overexpressing embryos

Partial rescue of NTDs resulting from excess *Grhl3* by deletion of the endogenous allele suggested that the deleterious effect of *Grhl3* overexpression on neural tube closure was localized to one or more of the endogenous sites of expression, as opposed to ectopic activity. At E8.5, *Grhl3* is expressed in the surface ectoderm and in the posterior part of the embryo corresponding to the node-streak border and caudo-lateral epiblast (11). At E9.5, *Grhl3* mRNA is also detected in the neural plate of the PNP and then, at E10–10.5, also in the hindgut endoderm. Whole mount *in situ* hybridization (WMISH)of *Grhl3*^*ct/ct*;*TgGrhl3/TgGrhl3*^ embryos confirmed *Grhl3* expression to be more intense but localized in the same tissues as in wild-type and hemizygous *Grhl3*-transgenic embryos (Fig. 4A and B) (11,12). Analysis of *Grhl3* expression in the context of lack of the endogenous allele further confirmed expression of the transgene in the normal expression domain (Fig. 4C).

In *Grhl3^-/-^* embryos, the timing of closure failure and the phenotype of conditional knockouts suggest that the earliest abnormality of spinal neural tube closure results from a defect in the surface ectoderm (12). The surface ectoderm is the precursor of the epidermis, in which *Grhl3* regulates terminal differentiation, barrier formation and repair (10,19–21). We asked whether expression of epithelial/epidermal genes was altered at neurulation stages. Using qRT-PCR, we found that known *Grhl3* targets in late-fetal epidermis, *Lor* and *Tgm1*, were already upregulated in the caudal region of *Grhl3* overexpressing embryos at E10.5 (Supplementary Material, Table S1). At E9, the stage at which neurulation begins to fail in *Grhl3* mutant embryos, *Trp63* (encoding TAp63) (22), a key transcriptional regulator of epidermal specification, was also upregulated in *Grhl3*^*ct/ct*;*TgGrhl3/TgGrhl3*^ (Supplementary Material, Table S2), suggesting abnormal specification of the surface ectoderm. This correlates with the finding that *Trp63* is downregulated in *Grhl3* null embryos at the same stage (12).

*Cdh1* (encoding E-cadherin) is a key marker of surface ectoderm, a direct target of *Grhl3* in mouse mammary gland cells (23) and upregulated in skin of *Grhl3*-transgenics at E18.5 (Supplementary Material, Fig. S6C). In the caudal region of *Grhl3*-transgenic embryos at E9.5 and 10.5, we observed a non-significant trend towards upregulation of *Cdh1* (Supplementary Material, Tables S1 and S2). As expression in the surface ectoderm comprises only a subset of the *Cdh1* expression domain we further examined expression by *in situ* hybridization at E8.5–10.5. Expression was observed at expected sites in the surface ectoderm and hindgut, with noticeably more intense staining in the surface ectoderm in *Grhl3*^*ct/ct*;*TgGrhl3/TgGrhl3*^ embryos compared with *Grhl3^ct/ct^* (Fig. 4D–E). Cells expressing *Cdh1* were also present at ectopic locations in the neuroepithelium of the PNP and tailbud of *Grhl3*^*ct/ct*;*TgGrhl3/0*^ and most notably *Grhl3*^*ct/ct*;*TgGrhl3/TgGrhl3*^ embryos (Fig. 4D–E and Supplementary Material, Fig. S3). Ectopic E-cadherin protein expression was also confirmed by immunohistochemistry (Supplementary Material, Fig. S3). We hypothesize that earlier overexpression of *Grhl3* in the node-streak border and caudo-lateral epiblast, which contains precursors of the neuroepithelium and mesoderm (neuromesodermal progenitors) (24), and in the neural plate may lead to persistent expression of *Cdh1*, such that expression is not downregulated in a subset of neuroepithelial cells. This idea is consistent with the observation of *Cdh1*-positive cells in ectopic locations within the tailbud. Given the sparse nature of *Cdh1*-expressing cells in the neural plate it seems unlikely that they have a significant deleterious effect on closure, particularly as elevation of the neural folds does not appear compromised in *Grhl3* overexpressing embryos.

The premature upregulation of markers such as *Trp63*, *Lor* and *Tgm1*, and the abnormal expression of *Cdh1*, suggest that NTDs caused by *Grhl3* gain of function could result primarily from dysregulation of gene expression in the surface ectoderm. A contribution from other Grhl3 expressing tissues appears unlikely as follows: overexpression of *Grhl3* in the neural plate occurs prior to closure failure but there is no obvious defect in neural fold elevation (Fig. 3). *Grhl3* is expressed in the node-streak border and caudo-lateral epiblast (Fig. 4A) but we found no evidence for altered patterning of the caudal region in *Grhl3* overexpressing embryos (Supplementary Material, Fig. S4). Loss of function of *Grhl3* in the hindgut alone is sufficient to prevent closure at late stages of spinal neurulation (12). However, in *Grhl3* overexpressing embryos, closure fails prior to onset of hindgut expression (Figs 3 and 4) and there is no increase in ventral curvature (Supplementary Material, Fig. S5), the mechanism by which insufficient *Grhl3* expression in the hindgut inhibits closure (13).

### Overexpression of *Grhl3* does not exacerbate cranial NTDs or cause skin barrier defects

In addition to SB, loss of function of *Grhl3* has been shown to cause cranial NTDs (exencephaly) and epidermal defects (9,10). We therefore asked whether overexpression of *Grhl3* recapitulates these phenotypes.

In *Grhl3^-/-^* models, the frequency of exencephaly was reported as 2% and 14% (9,10). Unlike SB this is not markedly higher than in the *curly tail* strain, in which exencephaly typically affects 6–8% of embryos (25–27). Among *Grhl3*^*ct/ct*;*TgGrhl3/0*^ intercrosses, exencephaly occurred in a similar proportion of transgene-negative *Grhl3^ct/ct^* (13/181; 7.2%) and transgene-positive (*Grhl3*^*ct/ct*;*TgGrhl3/0*^ and *Grhl3*^*ct/ct*;*TgGrhl3/Grhl3*^) embryos (39/487; 8.0%). Among 147 of these transgene-positive embryos genotyped by gPCR, the proportion of exencephalic embryos did not differ between BAC-hemizygotes and homozygotes. Hence, in contrast to the striking effect on SB, overexpression of *Grhl3* in the cranial region of *Grhl3*^*ct/ct*;*TgGrhl3/TgGrhl3*^ embryos (confirmed by qRT-PCR; Supplementary Material, Fig. S1C) did not cause a significant increase in frequency of cranial NTDs. Moreover, as we found previously, cranial NTDs in the *ct* strain were not rescued by upregulation of *Grhl3* in the cranial region of *Grhl3*^*ct/ct*;*TgGrhl3/0*^ embryos (27). These findings support the hypothesis that diminished *Grhl3* expression is not the main cause of cranial NTDs in the *ct* strain and that the underlying mechanism therefore differs from *Grhl3*-null embryos. Instead, the principal contribution may be from other deleterious variants in the *ct* genetic background, *Lmnb1* and *Mthfd1L* having been identified as potential modifiers of NTDs in the *ct* strain (25,27).

**Figure 5:**
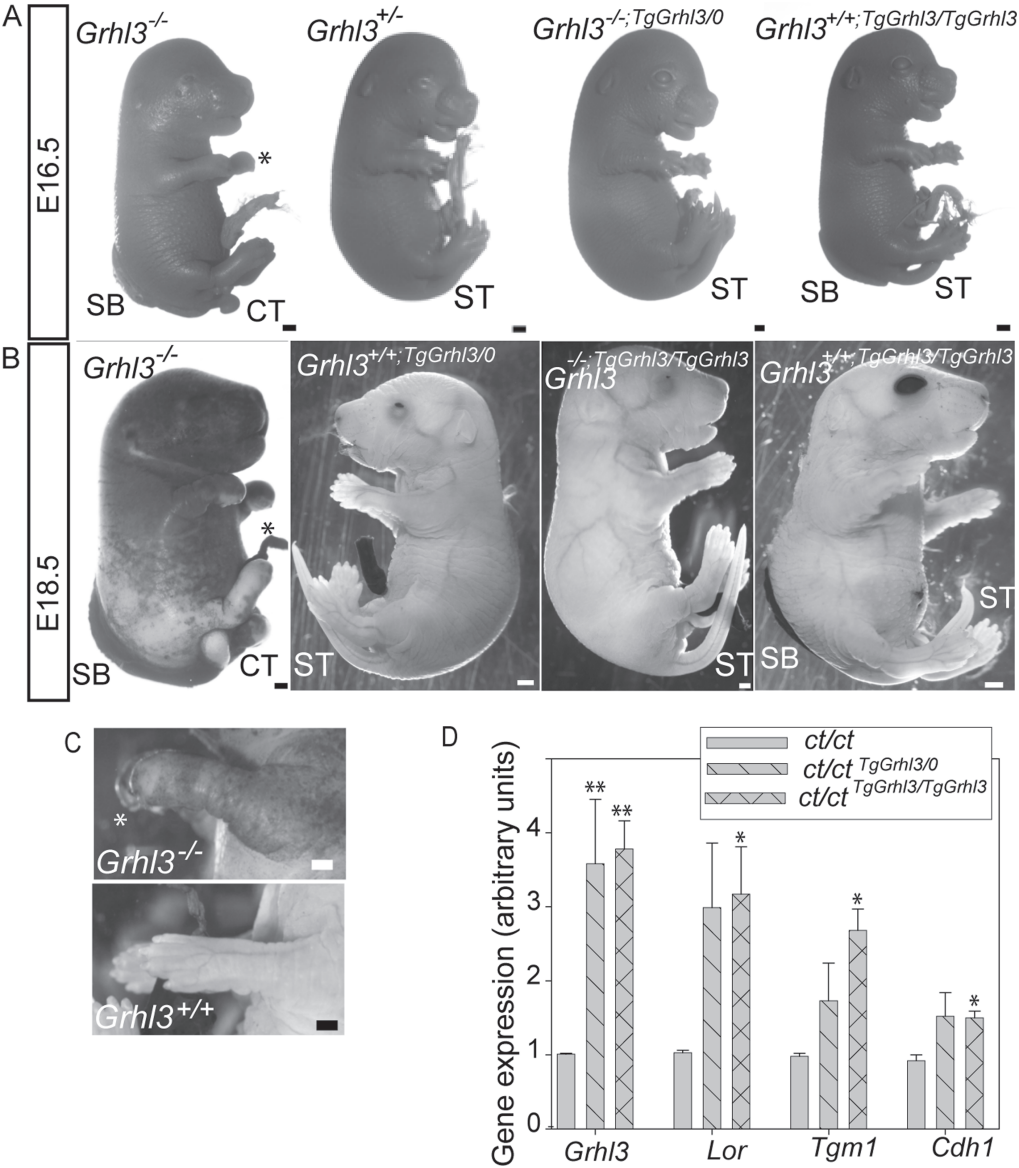
Establishment of the epidermal barrier is not compromised by *Grhl3* overexpression. (**A–C**) Integrity of the permeability barrier was determined by dye penetration assay. At E16.5 (A), all genotypes show dye penetration, whereas at E18.5 (B) *Grhl3*^-/-^ is the only genotype with incomplete epidermal barrier. Note that barrier formation is rescued by transgenic expression of *Grhl3* in *Grhl3*^*-/-*;*TgGrhl3/TgGrhl3*^ fetuses (in parallel with rescue of NTDs). Although *Grhl3* overexpression causes SB, the epidermal barrier is established by E18.5 in hemizygous and homozygous transgenics. Note that limbs appear normal in transgenic fetuses, unlike in *Grhl3^-/-^* (^*^, abnormal forelimbs in B,C). Moreover, partially or completely open eyelids are visible in the transgenic fetuses (B). Scale bar represents 1 mm; CT: curly tail; ST: straight tail. (**D**) Analysis of gene expression in E18.5 skin samples in litters from compound mutant/transgenic or *Grhl3*^*ct/ct*;*TgGrhl3/0*^ intercrosses shows that *Grhl3* overexpression is associated with increased expression of *Lor*, *Tgm1* and *Cdh1* (significant differences from *Grhl3^ct/ct^*: ^*^P < 0.05, ^**^P < 0.001; ANOVA).

*Grhl3* is required for differentiation and repair of the epidermal barrier at late fetal stages (10) and for post-natal repair of the epidermal barrier after injury (21). Skin histology of *Grhl3*-null fetuses becomes abnormal by E16.5, while the epidermal permeability barrier, which normally begins to form at E16–17, fails to develop by E18.5 (10). In the *Grhl3* null/transgenic crosses in the current study, we confirmed that the epidermal barrier fails to form in *Grhl3^-/-^* fetuses, using a dye penetration assay (Fig. 5A and B). The skin barrier was complete by E18.5 in 6/6 wild-type and 16/16 *Grhl3^+/-^* fetuses but not in *Grhl3^-/-^* littermates (0/5). Transgenic expression of *Grhl3* was sufficient to rescue the epidermal barrier defect phenotype in *Grhl3^-/-^* fetuses, both in hemizygous (*Grhl3*^-/-;TgGrhl3/0^) and homozygous (*Grhl3*^+/-;TgGrhl3/TgGrhl3^) transgenics (3/3 of each genotype tested) (Fig. 5). Known *Grhl3* targets in the epidermis were upregulated in skin at E8.5 (Fig. 5D). However, in contrast to the NTDs produced by overexpression of *Grhl3*, we did not observe epidermal abnormalities in *Grhl3*^*+/+*;*TgGrhl3*;*TgGrhl3*^ fetuses (barrier complete in 3/3; Fig. 5). The histological appearance of skin in *Grhl3*-overexpressing fetuses was also comparable to controls at E18.5, with normal staining for the basal marker p63 (Supplementary Material, Fig. S6).

Additional phenotypes reported in *Grhl3*-null fetuses include the presence of open eyelids at E18.5 and an abnormal limb phenotype (28). During normal development, the digits become separated by E15 and then undergo a temporary epithelial fusion, with displacement of intervening periderm cells (29). Digit fusion appears to occur normally in *Grhl3*-null fetuses but the distal limb appears swollen (28) (Fig. 5C). Consistent with normal barrier formation in *Grhl3* overexpressing embryos, limb development was also apparently normal (Fig. 5B). In contrast, the open-eye phenotype that accompanies SB in *Grhl3*-null fetuses was also present in *Grhl3* transgenics with SB at E18.5, whether wild type or mutant at the endogenous *Grhl3* locus.

### Genetic interaction of *Grhl3* and *Grhl2* overexpression alleles

**Figure 6:**
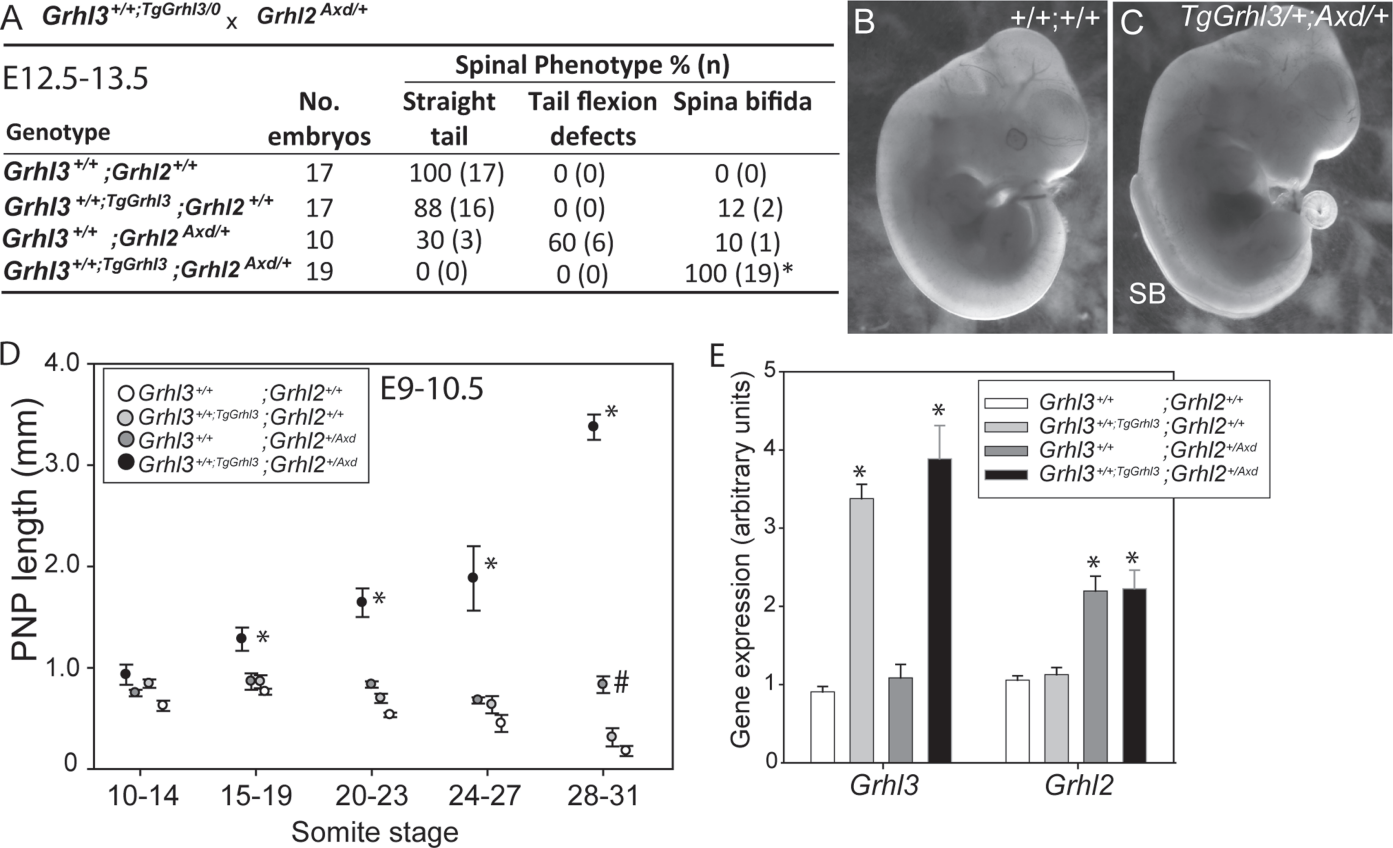
Genetic interaction between *Grhl2* and *Grhl3* overexpressing alleles results in severe SB. (**A–C**) Among experimental litters generated by intercross of *Grhl2^Axd/+^* and *Grhl3^+/+:TgGrhl3/0^* mice, all compound heterozygous fetuses exhibited severe SB. Note the very large size of the SB lesion that extends as far rostrally as the forelimb bud at E13.5 (C) (^*^P < 0.001; significant difference among genotypes, Chi-square). (**D**) Analysis of PNP length among embryos at E9–10.5 shows that compound heterozygous, *Grhl2^Axd/+^*;*Grhl3^+/+:TgGrhl3/0^* embryos have significantly enlarged PNPs from E9.5 (15–19 somite stage) onwards (^*^, significant difference from all other genotypes, *P* < 0.001). At E10.5 (28–31 somites) the PNP length of *Grhl2^Axd/+^*;*Grhl3*^*+/+*;*TgGrhl3/0*^ embryos is also greater than in wild type (^#^, *P* < 0.01). (**E**) In the caudal region of E9.5 embryos, mRNA abundance of *Grhl2* and *Grhl3* corresponds with the presence of the *Grhl2^Axd^* and *Grhl3^TgGrhl3^* alleles, with no indication of reciprocal regulation (^*^, differences from wild-type expression level, *P* < 0.01).

Grhl2 and Grhl3 both form homodimers but also exhibit protein–protein interactions to form heterodimers (30,31), although the functional role of these interactions is unknown. We speculated that excessive abundance of *Grhl3* could disturb the relative abundance of Grhl2 and Grhl3 proteins and favour formation of heterodimers, thereby inhibiting function of *Grhl2* homodimers and causing spinal NTDs (as observed in *Grhl2*-null embryos) (32). Similarly, it could be predicted that spinal NTDs caused by overexpression of *Grhl2* in *Axd* mutants could result from suppression of *Grhl3* function. These models predict that overexpressing *Grhl2* in *Grhl3*-transgenic embryos would normalize spinal neural tube closure by compensating for excess of Grhl3. To test this hypothesis we generated embryos that overexpress both *Grhl2* and *Grhl3*, by intercross of *Axd/+* and +/+^TgGrhl3/0^ mice. Spinal NTDs occurred only at very low frequency in single heterozygotes as expected (Fig. 6A). Remarkably, however, a large SB was observed in 100% (n = 19) of doubly heterozygous *Axd*/+; +/+^TgGrhl3^ embryos (Fig. 6A–C). Analysis of a further series of embryos collected at E9.0–10.5 showed that doubly heterozygous embryos exhibit failure of closure, with a significantly enlarged PNP from E9.5 (15–19 somites stage) onwards (Fig. 6D). We confirmed that expression of *Grhl2* and *Grhl3* was elevated in the embryos carrying the *Grhl2^Axd^* allele and *Grhl3* transgene, respectively (Fig. 6E), without evidence of reciprocal regulation. Hence, occurrence of SB results from an additive genetic interaction of the *Grhl2* and *Grhl3* alleles, and we can rule out a mechanism of NTDs based on mutual repression of function by gene overexpression.

### The *Vangl2^Lp^* allele genetically interacts with *Grhl3* overexpression to cause SB

We next asked whether moderate overexpression of *Grhl3* interacts with another genetic risk factor for NTDs, the *loop-tail* (*Lp*) mutation in *Vangl2*, encoding a core component of the PCP signalling pathway. Homozygosity for *Vangl2^Lp^* causes the severe NTD craniorachischisis, (33,34), while heterozygosity for *Vangl2* can also cause craniorachischisis or SB when in combination with mutant alleles of other genes including *Fzd2*, *Ptk7, Scrib* and *Sdc4* (35–37). Previous studies have also demonstrated genetic interaction of *Vangl2^Lp^* with the loss of function *Grhl3^ct^*- and *Grhl3*-null alleles (38,39). We intercrossed *Vangl2^Lp/+^* mice with *Grhl3*^*ct/ct*;*TgGrhl3/0*^ transgenics and analysed litters of embryos at E11.5 (Table 2). TFDs occurred in almost all *Vangl2^Lp/+^*;*Grhl3^ct/+^* double heterozygotes, correlating with the almost complete penetrance of this defect in *Vangl2* heterozygotes in the *loop-tail* strain (37). Strikingly, SB developed in 77% of *Vangl2^Lp/+^*;*Grhl3*^*ct/+*;*TgGrhl3/0*^ embryos, but not among embryos of any other genotype, demonstrating additive interaction of *Vangl2^Lp^* and the *Grhl3-*transgene (Table 2a).

**Table 2:** Genetic interaction between *Grhl3* and *Vangl2^Lp^* alleles results in severe SB

		**Spinal Phenotype % (n)**		
Genotype	**No. embryos**	**Straight tail**	**TFDs**	SB **& TFDs**
a. *Vangl2^Lp/+^* × *Grhl3^ct/ct TgGrhl3/0^*				
*Vangl2^+/+^*;*Grhl3^ct/+^*	4	100 (4)	0 (0)	0 (0)
*Vangl2^+/+^*;*Grhl3*^*ct/+*;*TgGrhl3/0*^	11	55 (6)	45 (5)	0 (0)
*Vangl2^Lp/+^*;*Grhl3^ct/+^*	12	8 (1)	92 (11)	0 (0)
*Vangl2^Lp/+^*;*Grhl3^ct/+ TgGrhl3^*	13	0 (0)	23 (3)	77 (10)*
b. *Vangl2^+/+^*;*Grhl3^ct/ct TgGrhl3/0^* × *Vangl2^Lp/+^*;*Grhl3^ct/+^*				
*Vangl2^+/+^*;*Grhl3^ct/+^*	14	86 (12)	14 (2)	0 (0)
*Vangl2^+/+^*;*Grhl3^ct/ct^*	11	55 (6)	45 (5)	0 (0)
*Vangl2^+/+^*;*Grhl3*^*ct/+*;*TgGrhl3/0*^	9	78 (7)	22 (2)	0 (0)
*Vangl2^+/+^*;*Grhl3*^*ct/ct*;*TgGrhl3/0*^	8	88 (7)	12 (1)	0 (0)
*Vangl2^Lp/+^*;*Grhl3^ct/+^*	6	0 (0)	100 (6)	0 (0)
*Vangl2^Lp/+^*;*Grhl3^ct/ct^*	9	0 (0)	22 (2)	78 (7)
*Vangl2^Lp/+^*;*Grhl3*^*ct/+*;*TgGrhl3*^	11	0 (0)	82 (9)	18 (2)
*Vangl2^Lp/+^*;*Grhl3*^*ct/ct*;*TgGrhl3*^	11	0 (0)	55 (6)	45 (5)

**(a)** Among experimental litters generated by intercross of *Grhl3*^*ct/ct*;*TgGrhl3/0*^ with *Vangl2^Lp/+^*, SB occurred at high frequency in embryos carrying both the *Grhl3* transgene and the *Vangl2^Lp^* allele, but was not observed among embryos carrying either allele alone (*P < 0.05; Chi square). **(b)** Embryos that are heterozygous or homozygous for the *Grhl3^ct^* allele but are wild type for *Vangl2* display partially penetrant TFDs. Doubly heterozygous (*Vangl2^Lp/+^*;*Grhl3^ct/+^*) embryos display TFDs, correlating with 100% penetrance of this defect among *Lp/+* mice. SB occurs with high frequency among *Lp/+*;*Grhl3^ct/ct^* embryos as reported previously (38). Unlike our finding in *Grhl3^ct/ct^* (Gustavsson et al., 2007) and *Grhl3^-/-^* embryos (Table 1), this defect is not rescued by the *Grhl3* transgene supporting the hypothesis that SB in embryos carrying the *Vangl2^Lp^* and *Grhl3* transgene, results from an additive genetic interaction. Litters analysed at E11.5 (n = 6 and 9 litters in a and b, respectively).

In a second experimental cross, *Vangl2^Lp/+^*;*Grhl3^ct/+^* double heterozygotes were intercrossed with *Grhl3*^*ct/ct*;*TgGrhl3/0*^ transgenics (Table 2b). Among litters analysed at E11.5 a high frequency of SB occurred in *Vangl2^Lp/+^*;*Grhl3^ct/ct^* embryos, confirming previous findings (38). However, in contrast to the preventive effect of hemizygosity for the *Grhl3*-BAC on NTDs in *Grhl3^ct/ct^* and *Grhl3^-/-^* embryos (Table 1), the presence of the *Grhl3*-transgene did not prevent the NTDs in *Vangl2^Lp/+^*;*Grhl3^ct/ct^* embryos (Table 2b). These findings provide further evidence for an exacerbating combinatorial effect of *Vangl2* mutation and *Grhl3* overexpression on spinal neural tube closure. This does not appear to involve direct regulation of *Vangl2* expression by Grhl3 as altered *Vangl2* expression was not associated with *Grhl3* loss of function in our previous transcriptomic analyses (10,21,27) or in qRT-PCR analysis of *Grhl3^+/+^* and *Grhl3^-/-^* embryos at E9.5 (*P* > 0.05; three or more per genotype). Similarly, *Vangl2* expression did not differ between *Grhl3^ct/ct^*, *Grhl3*^*ct/ct*;*TgGrhl3/0*^ or *Grhl3*^*ct/ct*;*TgGrhl3/TgGrhl3*^ embryos at E9.5 (*P* > 0.05; three or more per genotype). Moreover, Grhl3 binding sites were not identified in the 10 kb region upstream of *Vangl2* (39).

## Discussion

A requirement for sufficient *Grhl3* expression to enable spinal neural tube closure is shown by the presence of SB in *Grhl3*-null embryos (10), hypomorphic *Grhl3^ct/ct^* embryos (11) and in tissue-specific knockouts (12). In the current study we found that overexpression of *Grhl3* also causes SB, emphasizing the exquisite sensitivity of the closure process to the abundance of Grhl3 (Table 3). The induction of SB by excess *Grhl3* appears to involve a defect in the surface ectoderm: PNP closure fails soon after initiation of closure when *Grhl3* is strongly expressed in the surface ectoderm bordering the PNP and is not associated with a defect of neural fold elevation or altered molecular patterning of the caudal region. Moreover, *Grhl3* overexpressing embryos do not exhibit the excess body curvature that results from *Grhl3* loss of function in the hindgut and prevents the later stages of PNP closure (12).

**Table 3:** Summary of the relationship between spina bifida (SB) frequency and *Grhl3* abundance among embryos carrying different combinations of endogenous *Grhl3* allele and *Grhl3* transgene

Genotype & NTDs											
*Grhl3 locus*	*-/-*	*ct/-*	*ct/ct*	*+/-*	*+/+*	*ct/-*	*ct/ct*	*+/+*	*-/-*	*ct/-*	*ct/ct*
*Grhl3 transgene*	*0/0*	*0/0*	*0/0*	*0/0*	*0/0*	*Tg/0*	*Tg/0*	*Tg/0*	*Tg/Tg*	*Tg/Tg*	*Tg/Tg*
SB (%)	**100**	**50**	**10**	**0**	**0**	**0**	**0**	**15**	**38**	**70**	**70**
Increasing *Grhl3* expression →											
*Grhl3* expression	0		∼0.5	∼0.5	1		∼1.5	∼2			2.5-3

Indicative expression levels (approximate) are based on collation of qRT-PCR data within differing experimental crosses. The frequency of spina bifida (SB) refers to approximate percentage of embryos with SB among each specific genotype.

The *Grhl3* transgene is expressed in the endogenous domain during neural tube closure and rescue of SB by the presence of the transgene in *Grhl3^ct/ct^* and *Grhl3^-/-^* embryos shows that the target genes necessary for neurulation are appropriately regulated. Similarly, rescue of skin barrier defects shows that key epidermal genes are activated in the correct spatiotemporal manner. *Grhl3* is known to regulate numerous genes in the epidermis (derived from the surface ectoderm) at late-fetal and post-natal stages, including cell adhesion molecules, lipid metabolizing enzymes and structural proteins of the stratum corneum (10,19–21).

The genome-wide binding pattern of *Grhl3* appears dynamic with regulation of distinct sets of genes depending on the functional state of the epidermis. For example, comparison of epidermis during differentiation and during re-establishment of the epidermal barrier following post-natal injury shows overlap of Grhl3 targets, but more than half of the binding events differed (21). The potential for context-dependent and diverse transcriptional regulatory activity of Grhl3 suggests that the requirement during neural tube closure could involve overlapping and/or distinct functions compared with later epidermal differentiation and repair. Notwithstanding the possible difference in *Grhl3* targets at different stages, we found a subset of epidermal and epithelial markers to be upregulated in *Grhl3* overexpressing embryos at neurulation stages, suggesting a potential dysregulation of surface ectoderm properties. Such an effect could be incompatible with closure, consistent with the site of initial contact of the neural folds being mediated by surface ectoderm cells at the border with the neural plate (40).

The finding that both insufficient and excess abundance of Grhl3 cause SB is similar to observations with *Grhl2*, null alleles of which cause cranial and spinal NTDs (32,41,42), while upregulation causes SB (32). Interestingly, while *Grhl2*- and *Grhl3*-null embryos exhibit multiple defects alongside NTDs (e.g. skin barrier, urothelial differentiation, kidney and placental defects) (10,41,43,44), we found that their overexpression counterparts display isolated SB that more closely resembles the corresponding human condition.

Overall, findings in mouse models suggest that *GRHL2* and *GRHL3* represent strong candidates for potential involvement in human NTDs, with the consideration that not only loss-of-function variants but also regulatory and gain-of-function mutations could plausibly play a role. In addition to the development of NTDs in homozygous transgenic embryos it is notable that even moderate overexpression of *Grhl3* was sufficient to cause SB when in combination with heterozygous mutations in *Grhl2* or *Vangl2*. Such gene–gene interactions appear likely to more closely resemble the multigenic etiology of human NTDs than single gene mutants.

## Materials and Methods

### Mice

*Curly tail* mice were maintained as a homozygous, closed random-bred colony. The transgenic *curly tail* line *Grhl3^ct^/Grhl3^ct^*;Tg(*Grhl3*)1Ndeg (MGI:3794067), here denoted as *Grhl3*^*ct/ct*;*TgGrhl3/0*^, carries a BAC that encompasses the *Grhl3* gene (11). Homozygous transgenics were generated by intercross of *Grhl3*^*ct/ct*;*TgGrhl3/0*^ mice. The mutant line carrying a conditional (floxed) allele of *Grhl3* (designated *Grhl3^f/+^*) has been described (10). These mice were crossed to β-actin-*Cre* mice to generate heterozygous-null, *Grhl3*^+/-^, mice used in subsequent experimental matings. To generate embryos carrying combinations of *Grhl3* alleles with the *Grhl3-*BAC we crossed *Grhl3^+/-^* and *Grhl3*^*ct/ct*;*TgGrhl3/0*^ mice. The offspring with genotype *Grhl3*^*ct/-*;*TgGrhl3/0*^ were intercrossed to generate experimental litters.

To transfer the *Grhl3-*BAC onto a partial BALB/c genetic background, *Grhl3*^*ct/ct*;*TgGrhl3/0*^ mice were backcrossed with wild-type BALB/c mice for three generations. *Grhl3*^*+/+*;*TgGrhl3/0*^ were either intercrossed to generate experimental litters or crossed with *Axd/+* mice on a BALB/c genetic background (32), to generate litters containing double heterozygotes. Mice carrying the *loop-tail* allele of *Vangl2* were on a CBA/Ca background.

Animal studies were carried out under regulations of the Animals (Scientific Procedures) Act 1986 of the UK Government, and in accordance with guidance issued by the Medical Research Council, UK in *Responsibility in the Use of Animals for Medical Research* (July 1993). Mice were used for experimental matings from 6 weeks of age. Litters were generated by timed matings in which mice were paired overnight and the day of finding a copulation plug was designated embryonic day 0.5 (E0.5). Pregnant females were killed by cervical dislocation. The uterus was removed and transferred to Dulbecco’s Modified Eagles Media (DMEM; Invitrogen) containing 10% fetal bovine serum (heat inactivated at 58°C; Invitrogen).

The PNP length of embryos at E8.5–E10.5 was measured using an eyepiece graticule. The PNP was defined as the distance from the tip of the tail bud to the rostral limit of the open neural folds. For measurements of ventral curvature, the caudal region of E9.5–10.5 embryos was isolated from the body, photographed, and curvature of the caudal region measured as described (13,32). Briefly, as shown in Supplementary Material, Figure S6, the angle was determined in side views of the caudal region between a line tangential to the ventral edge of the penultimate somite and a line drawn along the midline of the tail bud, parallel to and equidistant from the ventral and dorsal surfaces.

### Genotyping

Mice were genotyped by PCR of genomic DNA, as described (10, 11,45). Mice carrying the *Grhl3* BAC-transgene were genotyped by PCR using a BAC-specific primer (11) and gene dosage determined by quantitative real-time genomic PCR (qG-PCR) on DNA isolated from individual yolk sacs amplified using RealTime PCR Mesa Blue qPCR Master Mix Plus for SYBR assay (EUROGENTEC), or iTAQ Universal SYBR Green Supermix assay (Bio-Rad). A *Grhl3* intronic sequence was amplified with normalization to a reference gene (*Grhl2*), whose copy number is unchanged between strains. Each assay experiment included at least one *Grhl3^ct/ct^* sample as calibrator and one hemizygous transgenic sample, for which the genotype was known (BAC-positive embryos from crosses between *Grhl3^ct/ct^* and *Grhl3^TgGrhl3/0^* mice). These samples provided an index level for the copy number of *Grhl3* in hemizygous transgenic, for comparison with the ‘unknown’ hemizygous or homozygous transgenic BAC-positive samples. PCR amplification of the transgene appeared more efficient than the endogenous *Grhl3* locus such that relative signal was 1 (2 endogenous copies), 2 (2 endogenous + 1 transgene) and 3 (2 endogenous + 2 transgene) (Fig. 3C).

### BAC localization by inverse PCR

DNA was extracted from transgenic embryos using the QIAamp DNA Mini kit (Qiagen), digested with *RsaI* (Invitrogen) or *HaeIII* (Fermentas), circularized and re-linearized with *BamHI* (Promega). The linearized product was then amplified by PCR with BAC inverse primers (327D13-R1 inverse_5′-CCCTAATGATGACCACGTGA-3′ and pTARBAC-R inverse_5′-TAGTGTCACCTAAATGTCGAC-3′). PCR products were separated on a 1% agarose gel and all obvious bands were excised from the gel and purified using QIAquick Gel Extraction kit (Qiagen). DNA was eluted in water for subsequent sequencing with each of the inverse primers (BigDye Terminator Cycle Sequencing kit, Applied Biosystems).

Sequence tags did not show 100% identity with a specific chromosomal location owing to variation between the unique *curly tail* genetic background and the C57BL/6 reference sequence. However, sequences generated from inverse PCR were aligned to the reference genomic sequence, with closest homology to a region on chromosome 18 that indicates insertion of the BAC at 18: 3,005,382 in a low complexity repeat. A series of primers (R1–R5; Supplementary Material, Table S5) complementary to chromosome 18 were used to amplify genomic DNA with the BAC-specific primer (pTARBAC-Rinv) (Supplementary Material, Fig. S1). To confirm localization genomic PCR was performed using a BAC specific primer (pTARBAC-R inverse) with a series of primers located in chromosome 18 (Supplementary Material, Table S4).

### Preparation of blood cultures and FISH

Interphase nuclei were prepared on slides using peripheral blood from *curly tail* and hemizygous *Grhl3* transgenic male mice. FISH analysis was performed on DAPI-stained interphase nuclei spreads on slides according to standard procedures using the BAC probe RP24-327D13 (BACPAC Resources Center at Childrens Hospital Oakland Research Institute).

Whole mount *in situ* hybridization was performed as previously reported (25). For sectioning, *embryos were* embedded in albumin-gelatine and 40 μm sections obtained on a vibratome (Leica VT 1000S, Leica Microsystems). Photography of whole embryos was performed on a stereo microscope (Leica MZFLIII microscope) using a Leica DC500 camera. Bright-field image acquisition of sections was performed with an Axiophot 2 microscope (Zeiss) with Leica DC500 camera software (AxioVision). Images were processed using Photoshop (Version 6.0) for cropping and figures were prepared using Adobe Illustrator software.

*Cdh1-*positive cells were counted on serial 40 μm sections after WMISH for *Cdh1*. For each embryo the total number of ectopic cells was divided by the total number of sections. The mean and the standard error of the mean (mean ± SEM) were plotted for different genotypes. To analyse the axial distribution of *Cdh1* positive cells in the neuroepithelium, four regions were defined and for each region the total number of positive cells per section was calculated as a percentage of the total number of sections (all embryos combined at each stage).

### Whole-mount antibody staining

4% PFA-fixed, methanol-dehydrated whole E8.5–E9.0 embryos were post-fixed (methanol/DMSO) overnight at 4°C. After bleaching (methanol/DMSO/30% H_2_O_2_) at room temperature for 4 h, embryos were blocked (phosphate buffered saline (PBS) containing 10% heat-inactivated Sheep serum/2% Bovine Serum Albumin/0.5% Triton X-100) for 4 h. Primary purified mouse anti-Ecadherin (BD Transduction Lab, 1:150 dilution) and relevant secondary (Alexa Fluor 488 goat anti-mouse, Life Technologies, 1/500) antibodies were applied in the same blocking solution overnight at 4°C. All the washes were performed with PBS 0.5% Triton X-100. Embryos were counterstained with DAPI for 2 h at room temperature. Prior to imaging, embryos were incubated in Scale A2 for clearing the tissue. Embryos were positioned in ‘wells’ cut into 4% agarose gels such that the PNP faced upwards. All images were captured on a Zeiss Examiner LSM880 confocal microscope using a 20x/NA1.0 Plan Apochromat dipping objective immersed in PBS. Low-magnification images were captured at 0.7× zoom with a pixel size of 0.6 μm and a Z step of 2 μm. High-magnification images were taken at 2× zoom with a pixel size of 0.1 μm and a Z step of 2 μm. Salt and pepper noise was subtracted, brightness and contrast were adjusted evenly across each image and maximum intensity projections were obtained in Fiji (ImageJ, NIH, *PMID 22743772*). Digital reslicing of confocal Z-stacks was also performed in Fiji, as previously (46).

### Quantitative real-time PCR

RNA was isolated from the caudal region of E8.5 (10–14ss, cut at somite 10), E9.5 (15–16ss, cut at somite 12) and E10.5 (26–31ss, cut at somite 14) embryos, and the cranial region, rostrally from the level of the otocyst (excluding branchial arches). Total RNA was isolated using TRIzol Reagent (Gibco) followed by DNase treatment (DNA-free, Ambion). cDNA was generated using the SuperScript VILO kit (Invitrogen) or SuperScript II Reverse Transcriptase (RT) kit (Invitrogen). Normalization was performed using glyceraldehyde-3-phosphate dehydrogenase (*Gapdh*) as (11). Quantitative RT-PCR was performed using iTAQ Universal SYBR Green Supermix assay (Bio-Rad) on a CFX96 system (Bio-Rad) with analysis using Bio-Rad CFX Manager software (see Supplementary Material, Table S5 for primer sequences). For each experiment a calibrator sample was chosen to normalize levels of cDNA expression. Individual experiments were combined and analysed using SigmaStat v 3.5 software (ANOVA or *t*-test).

## Supplementary Material

Supplementary Data
