# Supplementary material for: Overexpression of Grainyhead-like 3 causes spina bifida and interacts genetically with mutant alleles of Grhl2 and Vangl2 in mice
Source: Hum Mol Genet. 2018 Sep 4;27(24):4218–30. doi: 10.1093/hmg/ddy313 (PMC6276835; doi:10.1093/hmg/ddy313)
Supplement: Supplementary Data [file ddy313_supp.pdf]

SUPPLEMENTAL DATA

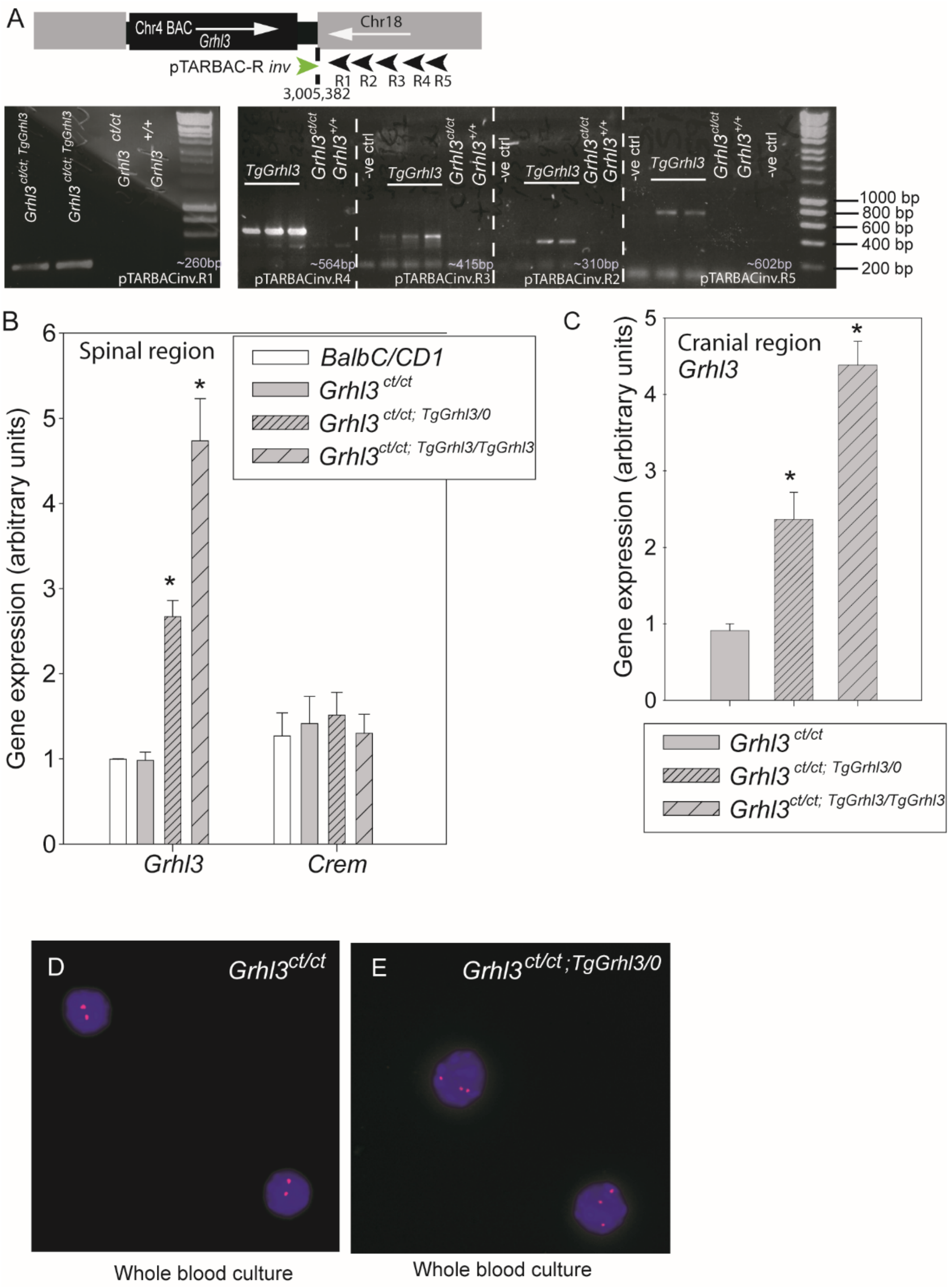

Figure S1. Inverse PCR to determine Grhl3-BAC insertion site

(**A**) Top: sequence tags most closely aligned to the reference genomic sequence in a region on chromosome 18 with insertion of the BAC at 18: 3,005,382. Bottom: in combination with a BAC-specific primer (pTARBAC-Rinv), PCR primers (R1 – R5), complementary to chromosome 18, amplified genomic DNA with bands of predicted sizes in transgenic (BAC-positive) samples but not in *Grhl3*$^{ct/ct}$ or wild-type (*Grhl3*$^{+/+}$) DNA. (**B**) qRT-PCR analysis

of the spinal region of E9.5 embryos (n = 3-5 of each genotype) found no effect of the *Grhl3*-BAC (*TgGrhl3*) on expression of *Crem*, the nearest gene (266 kb) from the BAC insertion site. As expected *Grhl3* was up-regulated in the same set of samples, in a dose-dependent manner, in hemizygous and homozygous transgenics (*P<0.001, ANOVA). (**C**) Similarly, *Ghrl3* is up-regulated in the cranial region of $Grhl3^{ct/ct;TgGrhl3/o}$ and $Grhl3^{ct/ct; TgGrhl3/TgGrhl3}$ embryos at E9 (12-13 somite stage; n = 4 of each genotype)(*p<0.001; TgGrhl3/0 significantly greater than *ct/ct* and TgGrhl3/TgGrhl3 significantly greater than both other genotypes). (**D,E**) FISH on interphase cells using a *Grhl3* probe produced two signals in $Grhl3^{ct/ct}$ cells as expected (D) and three signals in hemizygous transgenics ($Grhl3^{ct/ct; TgGrhl3/o}$)(E), consistent with a single integration site for the BAC.

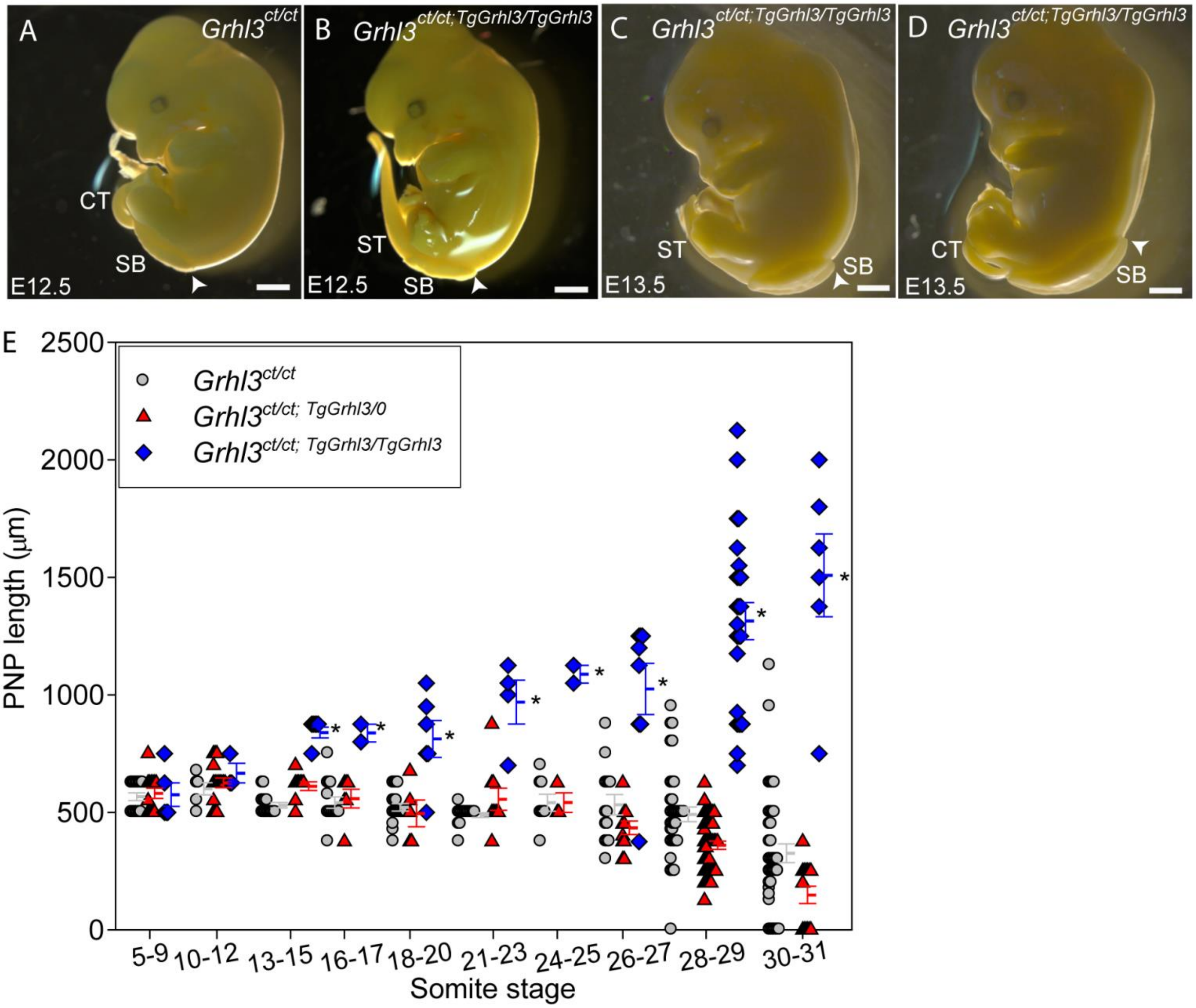

Figure S2. Spina bifida in *Grhl3* hypomorphic (*curly tail*) and over-expressing embryos

(**A-D**) The open spina bifida (SB) lesion does not extend as far rostrally in (A) *Grhl3*$^{ct/ct}$ embryos as in (B-D) *Grhl3*$^{ct/ct;TgGrhl3/TgGhrl3}$ (arrowhead indicates rostral extent of open lesion). Note that spina bifida (SB) in *Grhl3*$^{ct/ct;TgGrhl3/TgGhrl3}$ embryos frequently occurs without affecting tail curvature (B,C)(ST, straight tail; CT, tail flexion defect). Scale bars represent 1 mm. (**E**) PNP length plotted against somite number in *Grhl3*$^{ct/ct}$ embryos with 0, 1 or 2 copies of the *Grhl3*-BAC. Homozygosity for the *Grhl3* transgene produces a significantly enlarged PNP from the 16-17 somite stage onwards (* $p<0.05$). *Grhl3*$^{ct/ct}$ and *Grhl3*$^{ct/ct;TgGrhl3}$ embryos do not differ significantly in PNP length although a proportion of *Grhl3*$^{ct/ct}$ embryos show enlarged PNPs from the 26-27 somite stage onwards. These correspond to the *ct/ct* embryos that develop tail flexion defects and occasional SB (see Figure 1A).

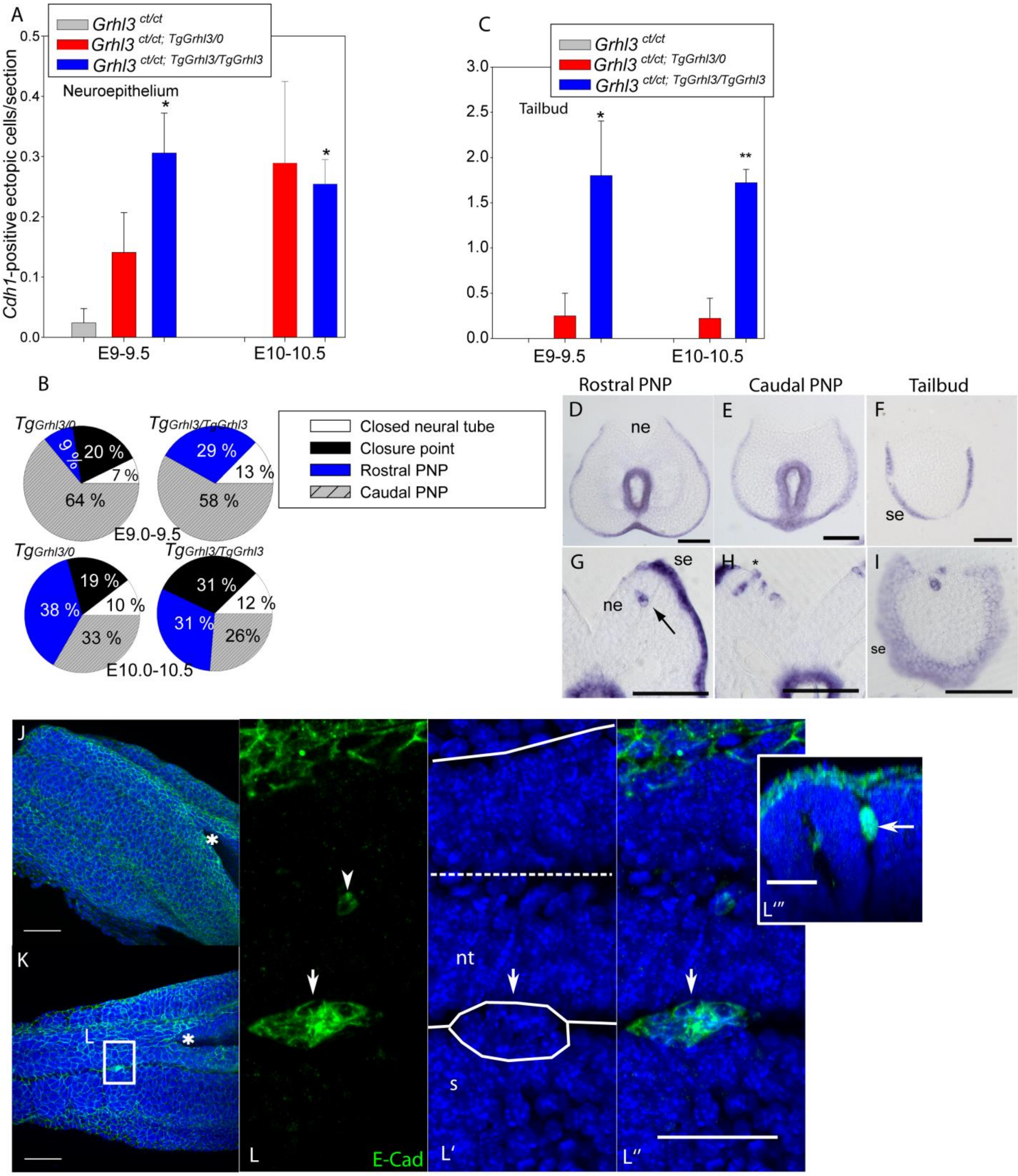

Figure S3. Ectopic *Cdh1* (E-cadherin) expressing cells in *Grhl3*-transgenic embryos.

(**A-C**) Quantification of the number of *Cdh1*-positive cells/clusters in transverse sections of the spinal region following whole mount *in situ* hybridisation for *Cdh1.* Neuroepithelium (A; combined regions as in B) and tailbud (C) show a significantly higher number of clusters in *Grhl3*$^{ct/ct;TgGrhl3/TgGrhl3}$ embryos compared with

Grhl3$^{ct/ct}$ (*p<0.01) at E9-9.5 (n = 5-7 embryos per genotype). At E10-10.5, *Cdh1* expressing cells are present in neuroepithelium of *Grhl3* transgenics but never in *Grhl3*$^{ct/ct}$ littermates (A) (n = 4 embryos per genotype). *Cdh1*-positive cells occur in the neuroepithelium throughout the open PNP (B) and in the immediately closed neural tube (data represent proportion of total *Cdh1*-positive cells observed). (**D-I**) Representative images of in situ hybridisation for *Cdh1* at E9-9.5 in the open PNP (rostral and caudal levels) and tail bud in *Grhl3*$^{ct/ct}$ (D-F) and *Grhl3*$^{TgGrhl3/TgGrhl3}$(G-I) embryos. Note *Cdh1*-expressing cells in surface ectoderm (se) of both genotypes, with occasional positive cells also in the neuroepithelium (ne) of *Grhl3*$^{TgGrhl3/TgGrhl3}$ embryos only (e.g. arrow in G). Most *Cdh1* positive cells appear to be integrated into the neuroepithelium but some appeared to be in the process of extrusion from the apical or basal surface of the neuroepithelium (* H). (**J,K**) Whole mount immunohistochemistry of *Grhl3*$^{ct/ct}$ and *Grhl3*$^{ct/ct;TgGrhl3/TgGrhl3}$ embryos at E9.0, stained for E-cadherin (green) and DAPI (blue). Single E-cadherin expressing cells or cell clusters are visible that are clearly distinct from the surface ectoderm. Asterisks: PNP closure point. (**L-L''**) Boxed region in K shown at higher magnification in E-cadherin (L), DAPI (L') and merged (L'') images. Small arrow indicates an E-cadherin positive cell cluster at the basal surface of the neural tube (nt), adjacent to the somitic mesoderm (s); arrowhead points to a positive cell adjacent to the neural tube lumen. (**L'''**) Digitally re-sliced rostro-caudal view of the embryo in K-L'', illustrating the position of the E-cadherin positive cluster (white arrow) between neural tube and somitic mesoderm, clearly separate from surface ectoderm. The single E-Cad positive cell is visible at the apical surface of the neuroepithelium (arrowhead in L). Scale bars represent 0.1 mm (D-I), 100 µm (J,K) and 50 µm (L). Abbreviations: nt, neural tube; s, somite; dashed line shows neural tube lumen; solid lines define nt/s boundary.

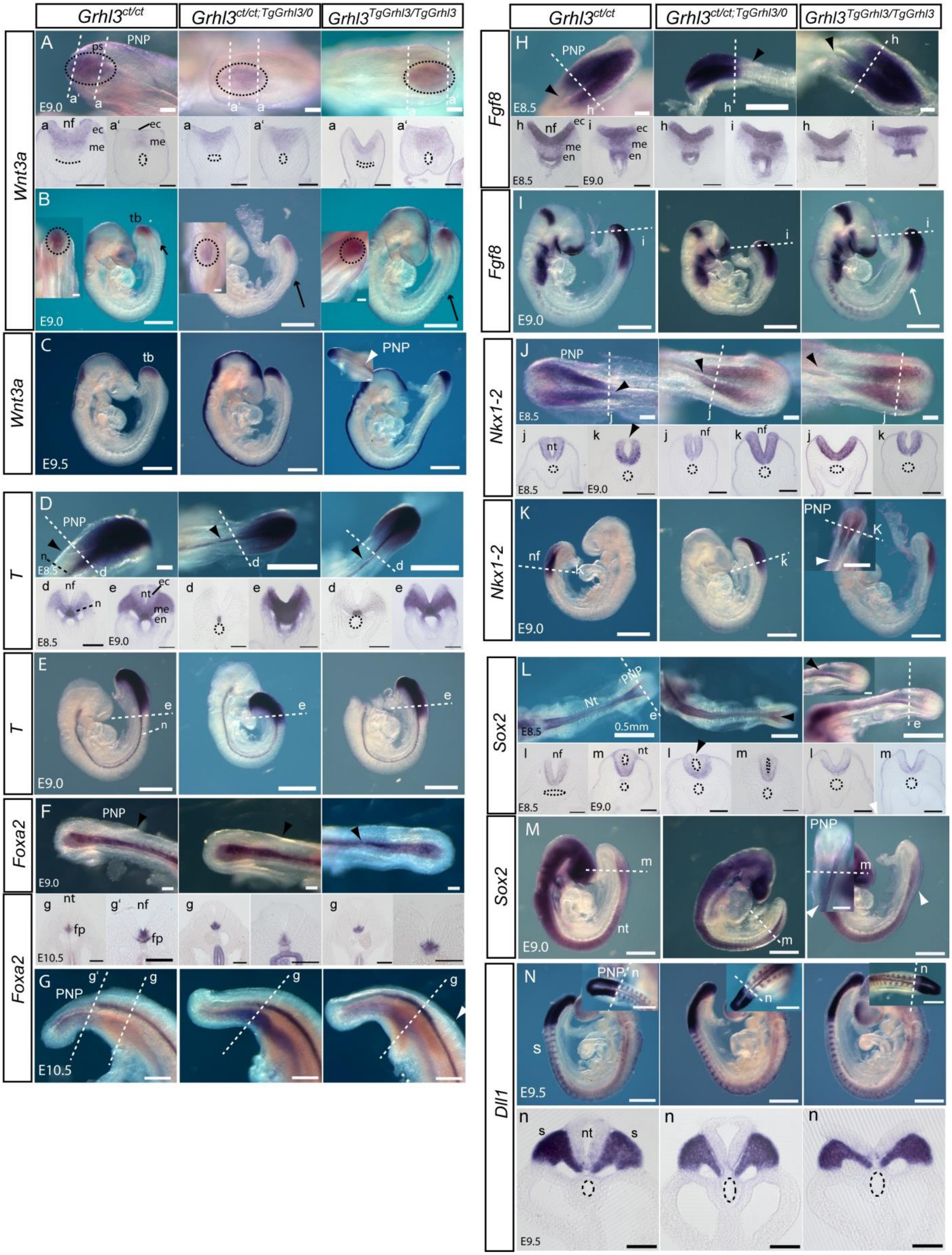

Grhl3ct/ct
Grhl3ct/ct;TgGrhl3/0
Grhl3TgGrhl3/TgGrhl3
Wnt3a
Wnt3a
T
T
Foxa2
Foxa2
Fgf8
Fgf8
Nkx1-2
Nkx1-2
Sox2
Sox2
Dll1
A
B
C
D
E
F
G
H
I
J
K
L
M
N
PNP
ps
nf
ec
me
en
nt
tb
fp
n
s
Nt
E8.5
E9.0
E9.5
E10.5
0.5mm

Figure S4. Expression of caudal genetic markers in *Grhl3* over-expressing embryos.

(**A-C**) *Wnt3a*, (**D-E**) *T*, (**F-G**) *Foxa2*, (**H-I**) *Fgf8*, (**J-K**) *Nkx1-2*, (**L-M**) *Sox2* and (**N**) *Dll1* were analysed by whole mount *in situ* hybridisation in *Grhl3*$^{ct/ct}$, *Grhl3*$^{ct/ct;TgGrhl3/0}$ and *Grhl3*$^{ct/ct;\ TgGrhl3/TgGrhl3}$ embryos at E8.5 (D, H, J, L), E9.0 (B, E, F, I, K, M) and/or E10.5 (G). Images in A, D, F, H, J show dorsal views of the caudal region. Arrowheads indicate anterior extent of open PNP. Dashed lines indicate level of sections in corresponding transverse sections. Intensity and anterior limit of expression of caudal markers (*Wnt3a*, *T*, *Fgf8*) does not differ between genotypes and occurs at the same axial level irrespective of the presence of an enlarged PNP. Expression of *Foxa2* in the ventral midline appears unaffected in embryos in which neural tube closure is disrupted, while specification of the neural plate as judged by expression of the early markers *Nkx1-2* and *Sox2* is not altered in *Grhl3* over-expressing embryos. Expression of *Dll1* in the caudal region and somites at E9.5 is closely similar between genotypes, suggesting that over-expression of *Grhl3* has no discernible effect on formation of paraxial mesoderm or somitogenesis. Scale bars represent 0.5 mm (embryos in A-N) and 0.1 mm (sections in a-n and inserts in B, K, N, L). Abbreviations: nf, neural folds; nt neural tube; s, somites.

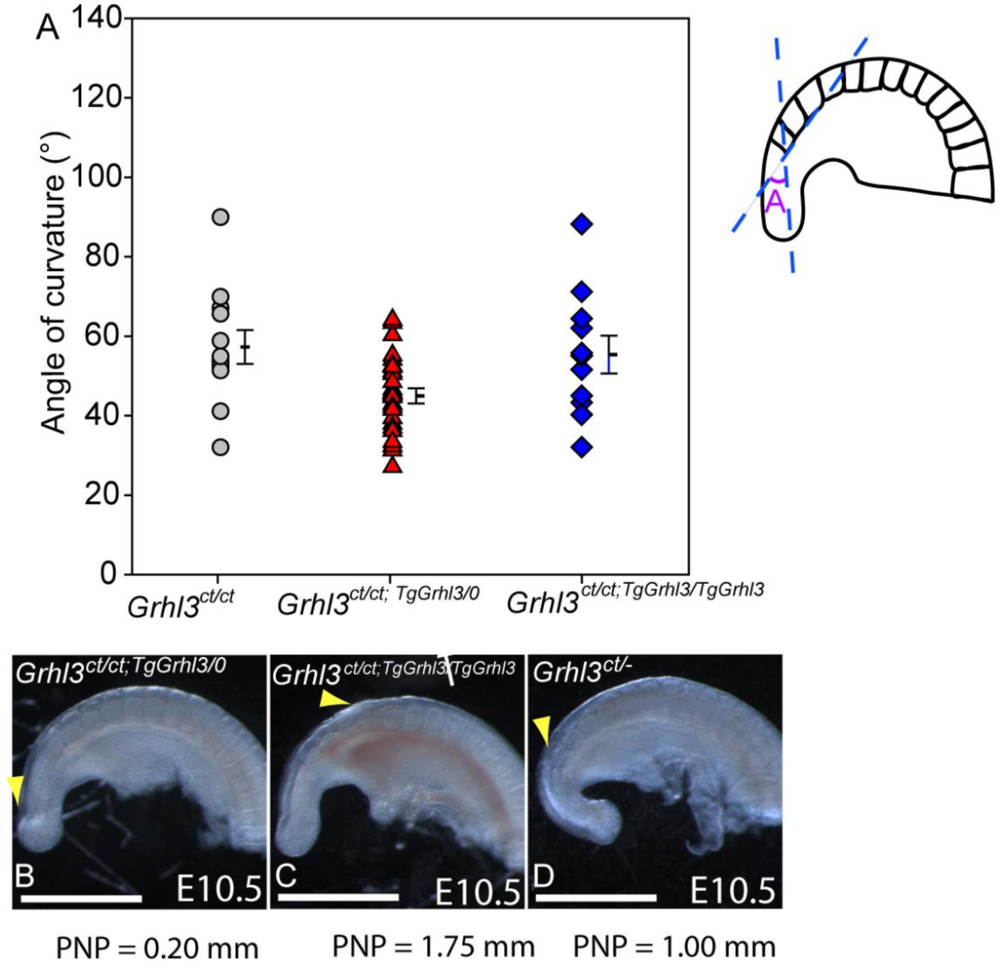

Figure S5. Curvature of the caudal body axis is not increased in Grhl3 over-expressing embryos.

(**A**) Angle of ventral curvature (indicated as A in diagram; see (12) does not differ between embryos of *Grhl3*$^{ct/ct}$, *Grhl3*$^{ct/ct;TgGrhl3/0}$ and *Grhl3*$^{ct/ct;\ TgGrhl3/TgGrhl3}$ genotypes at E10.5 (26-29 somite stage). (**B-D**) Isolated E10.5 caudal regions showing PNP lengths below the images. Note lack of increased ventral curvature in a *Grhl3*$^{ct/ct;\ TgGrhl3/TgGrhl3}$ embryo with enlarged PNP (rostral limit shown by yellow arrowhead in C) compared with *Grhl3*$^{ct/ct;TgGrhl3/0}$ embryo that has a PNP of normal length (B). As a positive control, *Grhl3*$^{ct/-}$ embryo with enlarged PNP (D) shows strongly enhanced ventral curvature. Scale bars represent 1 mm.

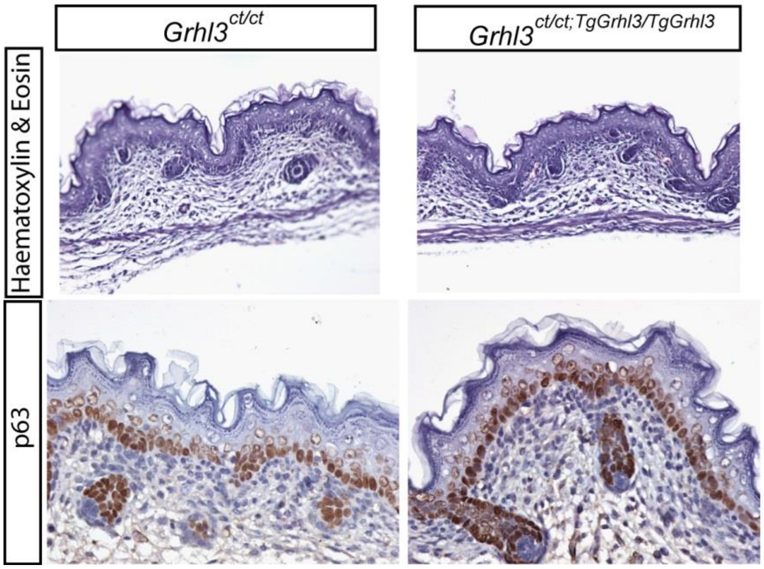

Figure S6. Histology and p63 immunostaining of skin at E18.5

Skin isolated from matched areas of the upper back of fetuses at E18.5 was fixed and sectioned followed by H&E staining or immunostaining for p63 (brown). No notable differences are observed between *Grhl3*$^{ct/ct}$ and *Grhl3*$^{ct/ct;\ TgGrhl3/TgGrhl3}$ samples.

Supplementary Tables

Genotype	*No. samples*	*Grhl3*	*Lor*	*Tgm1*	*Cdh1*
***Grhl3**$^{ct/ct}$*	6-12	**0.98±0.06**	1.05±0.04	0.79±0.13	1.00 ± 0.04
***Grhl3**$^{ct/ct;TgGrhl3/0}$*	5-11	**3.64±0.59****	1.32±0.03	**1.26±0.08***	1.01 ± 0.06
***Grhl3**$^{ct/ct;TgGrhl3/TgGrhl3}$*	7-11	**7.45±0.91****	**1.42±0.16***	**1.72±0.25***	1.15 ± 0.12

Table S1. Expression of known targets of *Grhl3* in epithelia or epidermis in the caudal region of *Grhl3* transgenic embryos at E10.5. mRNA abundance was determined by qRT-PCR for loricrin (*Lor*), transglutaminase 1 (*Tgm1*) and E-cadherin (*Cdh1*), with data normalised to the control (*Grhl3*$^{ct/ct}$) within each group. Significant differences from *Grhl3*$^{ct/ct}$ are observed for *Grhl3*, *Lor* and *Tgm1*, but not for *Cdh1* (**p<0.005, *p<0.05; ANOVA).

Genotype	*No. samples*	*Grhl3*	*Trp63* (p63)	*Cdh1* (E-cad)
***Grhl3**$^{ct/ct}$*	5-7	0.94 ± 0.06	1.03± 0.02	1.02±0.02
***Grhl3**$^{ct/ct;TgGrhl3/0}$*	6-13	**4.43 ± 0.34****	0.92± 0.08	1.10± 0.18
***Grhl3**$^{ct/ct;TgGrhl3/TgGrhl3}$*	5-9	**7.67 ± 0.70****	**1.30± 0.05****	1.18± 0.11

Table S2. Expression of epithelial/epidermal markers in the caudal region of *Grhl3* transgenic embryos at E9.0-9.5. mRNA abundance was determined by qRT-PCR with data normalised to a *Grhl3*$^{ct/ct}$ control. **significant difference compared with *Grhl3*$^{t/ct}$ (p<0.005; ANOVA and pairwise comparisons). Note that *Trp63* is significantly down-regulated in *Grhl3*$^{-/-}$ embryos at this stage (12).

	Somite Stage				
Genotype	10-14	15-19	20-23	24-27	28-31
***Grhl2**$^{+/+}$; **Grhl3**$^{+/+}$*	4	20	25	4	9
***Grhl2**$^{+/+}$; **Grhl3**$^{+/+;TgGrhl3/0}$*	9	30	20	5	6
***Grhl2**$^{Axd/+}$; **Grhl3**$^{+/+}$*	7	18	8	9	3
***Grhl2**$^{Axd/+}$; **Grhl3**$^{+/+;TgGrhl3/0}$*	7	16	21	7	3

Table S3. Number of embryos used for posterior neuropore (PNP) length measurements (Fig 6D).

Primer name	*Chromosomal region*	*Primer sequence*
Chr18_R1	18:3,005,603-3,005,621	5’-TGGCTGGCCTTCATGTGA-3’
Chr18_R2	18:3,005,653-3,005,671	5’-GTGCATGTCAGCTATCTTG-3’
Chr18_R3	18:3,005,757-3,005,776	5’-AACACAGAAGGGAGGTGGTG-3’
Chr18_R4	18:3,005,906-3,005,925	5’-AGAAACCACCATGCAGCTCT-3’
Chr18_R5	18:3,005,944-3,005,963	5’-ATATACCTGCATGCCAGGAG-3’

Table S4. Primers for BAC localisation. The genomic region within chromosome 18 and the primer sequences are shown.

Target gene	Primer sequence	
Grhl3	5’- ATGACAATGGCTCCCTCAAC	5’- GAGCCCAGGGTGTATTCAAA
Cdh1	5’-CCTGCCAATCCTGATGAAAT	5’-GTCCTGATCCGACTCAGAGG
Lor	5’- TACCTGGCCGTGCAAGTAAG	5’- ACAGGATACACCTTGAGCGAC
Tgm1	5’-CTCCTTCTGGGCTCGTTGTT	5’-ATTTACACCACTGCCCCGAG
Trp63	5’-CCTTATGAGCCACCACAGGT	5’-GCTGTCTTCATCTGCCTTCC
Vangl2	5’-CCGGGGATTGGGTAGCGTGT	5’-TGAAGGAGGTGGCTGTGGGACC

Table S5. Primers for qRT-PCR
